# Ethyl gallate isolated from phenol-enriched fraction of *Caesalpinia mimosoides* Lam. Promotes cutaneous wound healing: a scientific validation through bioassay-guided fractionation

**DOI:** 10.3389/fphar.2023.1214220

**Published:** 2023-06-16

**Authors:** Pradeep Bhat, Vishal S. Patil, Ashish Anand, Subhas Bijjaragi, Ganesh R. Hegde, Harsha V. Hegde, Subarna Roy

**Affiliations:** ^1^ Indian Council of Medical Research-National Institute of Traditional Medicine, Belagavi, India; ^2^ Post Graduate Department of Studies in Botany, Karnatak University, Dharwad, India; ^3^ Solid State and Structural Chemistry Unit, Indian Institute of Science, Bengaluru, India; ^4^ KLE’s SCP Arts, Science and D. D. Shirol Commerce College, Bagalkot, India

**Keywords:** *Caesalpinia mimosoides*, ethyl gallate, wound healing, isolation, phenols, antimicrobial, antioxidant

## Abstract

The tender shoots of *Caesalpinia mimosoides* Lam. are used ethnomedically by the traditional healers of Uttara Kannada district, Karnataka (India) for the treatment of wounds. The current study was aimed at exploring phenol-enriched fraction (PEF) of crude ethanol extract of tender shoots to isolate and characterize the most active bio-constituent through bioassay-guided fractionation procedure. The successive fractionation and sub-fractionation of PEF, followed by *in vitro* scratch wound, antimicrobial, and antioxidant activities, yielded a highly active natural antioxidant compound ethyl gallate (EG). *In vitro* wound healing potentiality of EG was evidenced by a significantly higher percentage of cell migration in L929 fibroblast cells (97.98 ± 0.46% at 3.81 μg/ml concentration) compared to a positive control group (98.44 ± 0.36%) at the 48th hour of incubation. A significantly higher rate of wound contraction (98.72 ± 0.41%), an elevated tensile strength of the incised wound (1,154.60 ± 1.42 g/mm^2^), and increased quantity of connective tissue elements were observed in the granulation tissues of the 1% EG ointment treated animal group on the 15th post-wounding day. The accelerated wound healing activity of 1% EG was also exhibited by histopathological examinations through Hematoxylin and Eosin, Masson’s trichome, and Toluidine blue-stained sections. Significant up-regulation of enzymatic and non-enzymatic antioxidant contents (reduced glutathione, superoxide dismutase, and catalase) and down-regulation of oxidative stress marker (lipid peroxidation) clearly indicates the effective granular antioxidant activity of 1% EG in preventing oxidative damage to the skin tissues. Further, *in vitro* antimicrobial and antioxidant activities of EG supports the positive correlation with its enhanced wound-healing activity. Moreover, molecular docking and dynamics for 100 ns revealed the stable binding of EG with cyclooxygenase-2 (−6.2 kcal/mol) and matrix metalloproteinase-9 (−4.6 kcal/mol) and unstable binding with tumor necrosis factor-α (−7.2 kcal/mol), suggesting the potential applicability of EG in inflammation and wound treatment.

## 1 Introduction

A wound is an injury that disrupts the anatomical arrangement of the cells and affects the integrity and functionality of the living skin tissue. Wounds are clinically classified as acute and chronic, on the basis of physiology and reparative time frame (Oluwole et al., 2022). Wound healing is an intricate physiological process with the temporal synchronization of a variety of cells, coordinating with four over-lapping phases *viz*. hemostasis, inflammation, proliferation, and tissue remodeling (Kasouni et al., 2021). The wound healing process is strictly regulated by the manifestation of various growth factors and cytokines at the wound site. Any impediments or alterations during the healing process can cause severe tissue damage and infections, which may harm the adjacent healthy tissues. Consequently, it prolongs the tissue repair process and leads to chronic or non-healing wounds, which can create severe clinical complications or even death (Amri et al., 2017; Negut et al., 2018).

Wound care facilities have gained tremendous attention throughout the world as they significantly affect the healthcare economy. A retrospective study of medicare recipients in 2018 revealed approximately 8.2 million people around the globe were suffering from contagious or non-contagious wounds, and the cost of treatment ranged between 28.1 and 96.8 billion dollars (Ustuner et al., 2019). In 2017–2018, the annual wound management cost in the United Kingdom was more than £5.6 billion (Oluwole et al., 2022), and around 5.7 million patients in the United States were suffering from chronic wounds with an annual treatment cost of $20 billion (Kumari et al., 2022). All these studies have demonstrated that there is a huge scope for research on the proper management of wounds with distinct modulatory effects.

In recent years, plant-based compounds and their products have shown improved wound healing through natural repair mechanisms by shortening the prolonged inflammatory phase, enhancing the migration and proliferation of fibroblasts, stimulating the process of angiogenesis, and thereby accelerating the re-epithelization process. Numerous studies on the potentiality of natural products as wound healing agents have scientifically evaluated their antimicrobial, antioxidant, and anti-inflammatory properties (Santos et al., 2021). In these circumstances, the World Health Organization (WHO) encourages the utilization of scientifically validated plant-derived drugs (World Health Organization, 2013).

Natural products from traditional medicines and ethnopharmacology are an important source of novel leads in the development of drug discovery research for therapeutic applications. In the past decades, pharmaceutical industries have been less attentive toward natural products, specifically due to their inherent complexities. However, recent technological advancements assisted in addressing new disputes to a great extent, and has rejuvenated the scientific interest in the field of drug discovery from natural resources (Najmi et al., 2022). The separation and isolation of active compounds from plant extracts with subsequent identification are important steps in the drug discovery process. Bioassay-guided fractionation and isolation is one such effective procedure to discover novel therapeutic compounds from active fractions of plant extracts. In this process, each fraction produced during chromatographic separation is evaluated for specific biological activities. Only the active fractions are further fractionated and re-fractionated until the isolation of a single or multiple pure biologically active compounds (Moyo et al., 2019; Muhammad et al., 2021).

*Caesalpinia mimosoides* Lam. is an important traditional medicinal plant that belongs to the family Fabaceae, native to Southeast Asia, and distributed in India, Thailand, Burma, Laos, Vietnam, and China (WFO, 2022). It is a sub-erect or scandent shrub, densely hispid, with recurved prickles throughout the plant. The tribal communities of Thailand traditionally consume tender shoots and immature leaves of the plant as a vegetable, appetizer, carminative, and to get rid of giddiness (Chanwitheesuk et al., 2007; Tangsaengvit et al., 2013; Manasa et al., 2014). The *Mullu Kuruma* tribe of Kerala state, India, uses plant leaves in treating epilepsy (Silja et al., 2008). During our ethnomedicinal exploratory studies in the Central Western Ghats region, Karnataka, India, we reported the use of *C. mimosoides* tender shoots in treating wounds and skin diseases (Bhat et al., 2012; Bhat et al., 2014). In addition, the traditional healers of the Udupi district of Karnataka use the root paste of the plant in treating wounds, ulcers, and arthritis (Rekha, 2011).

In our earlier studies, the wound-healing activity of crude ethanol extract of *C.mimosoides* was scientifically evaluated (Bhat et al., 2016). Further, phenolic enrichment of bioactive crude ethanol extract was performed through a sequential liquid-liquid partition process, and the resulting phenol-enriched fraction (PEF) was evaluated for wound-healing properties, followed by antioxidant, antimicrobial, and anti-inflammatory activities (Bhat et al., 2022). The effective wound-healing activity of PEF over crude ethanol extract has created an interest to take it for further bioassay-guided fractionation process to get a pure and biologically active compound, “ethyl gallate” (EG). In the present study, the fractions, sub-fractions, and the isolated compound EG were analyzed by *in vitro* toxicity and viability studies, followed by *in vitro* wound-healing activity through scratch wound assay against L929 cells. All the samples were further evaluated for *in vitro* antimicrobial activity against bacterial and fungal skin pathogens causing wound infections. In addition, the highly active fractions and sub-fractions were evaluated for *in vitro* antioxidant activity. *In vivo* acute toxicity and wound-healing activity of EG was carried out in Wistar albino rats, followed by histopathology and biochemical estimations of granulation tissues. *In silico* docking studies were also performed to elucidate the wound-healing mechanism of EG.

## 2 Materials and methods

### 2.1 Collection and extraction of plant material, and obtaining PEF

Healthy tender shoots of *C. mimosoides* were collected from the thick evergreen forests of Yana village (14° 34′ 0″ N and 74° 32′ 59″ E), located in the Central Western Ghats region of Uttara Kannada district, Karnataka, India. The plant was authentically identified using standard regional floras and the voucher specimen (PB/GRH-111) was deposited in the Herbarium, Karnatak University, Dharwad for future reference. The freshly collected tender shoots of the plant (2.5 kg) were shade dried and coarsely powdered with an electrical blender. The powdered material [1 kg dry weight (d.w.)] was sequentially extracted with the solvents (n-hexane, chloroform, acetone, ethanol, and water) of increasing polarity and dielectric constants through the hot percolation method using Soxhlet apparatus (Bhat et al., 2016). The active ethanol extract with potent wound-healing activity was further processed for the enrichment of phenols using liquid-liquid partition procedure as described in our earlier publication (Bhat et al., 2022). The resulting PEF was further dried using a rotary evaporator (Heidolph Laborota 4000), with a yield of 28.90 ± 0.64% (d.w.), and it was stored at 4°C in a refrigerator for further experimental use.

### 2.2 Bioassay-guided fractionation of PEF through chromatographic techniques and isolation of an active constituent

**FIGURE 1:**
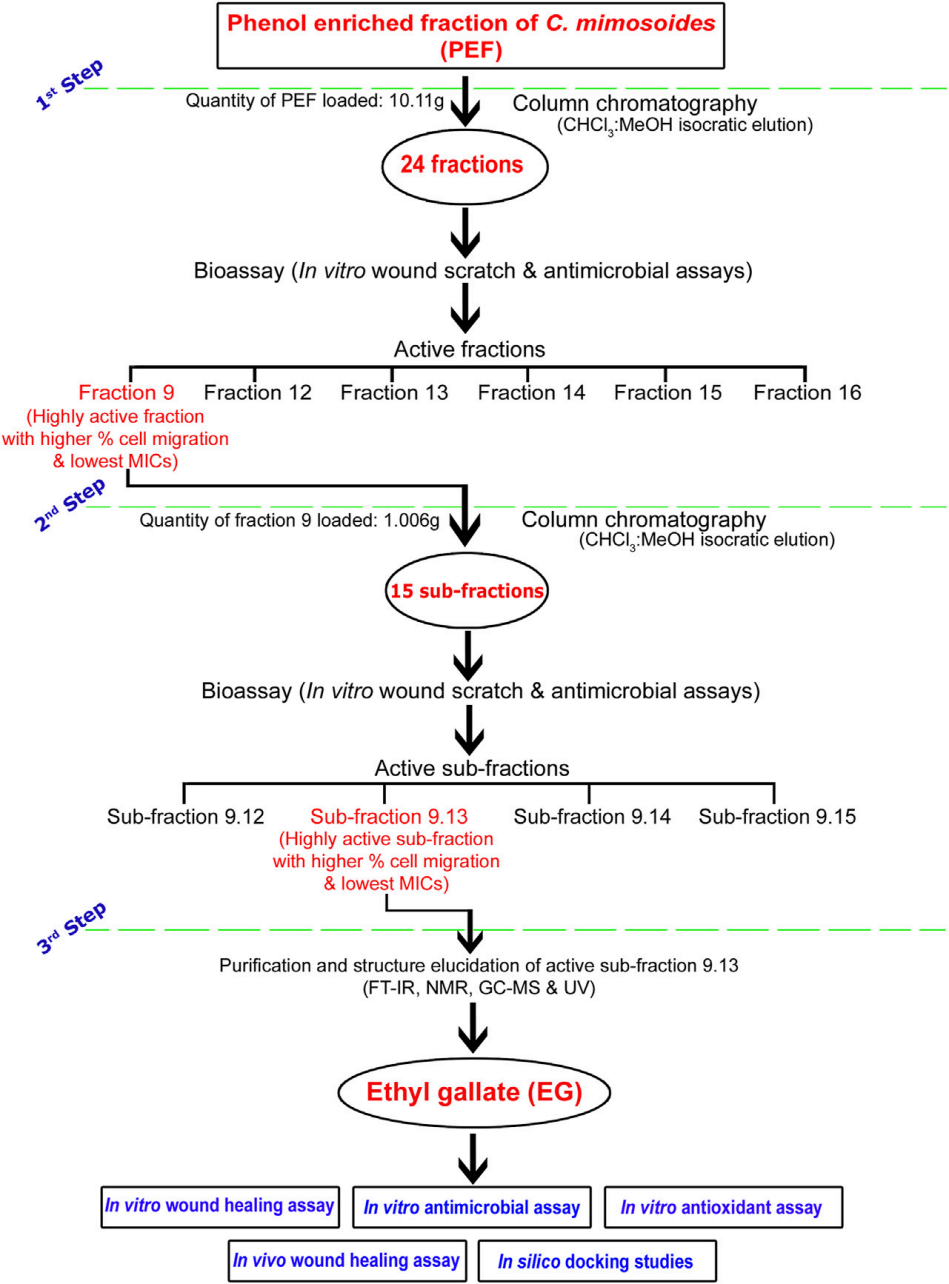
Flow chart representing brief methodology of bioassay-guided fractionation, isolation, and purification of biologically active compound EG.

A flow chart representing a brief methodology of bioassay-guided fractionation, isolation, and purification of the biologically active compound EG is given in Figure 1. A chromatography glass column (size: 60 cm × 30 mm) with an integral sintered disc (JAIN Scientific Glass Works, Haryana, India) was used for the fractionation process. The column was packed with activated silica gel (mesh size 60–120) by mixing chloroform through the slurry method (Özbilgin et al., 2018). The dried PEF (10.11 g) and the activated silica gel (mesh size 60–120) dissolved in chloroform were loaded on the top of the silica bed in the column. The isocratic mode of elution was performed with CHCl_3_, combinations of CHCl_3_:MeOH (99.80:0.20; 99.50:0.50; 99:1; 98.50:1.50; 98:2; 97.50:2.50; 97:3; 96.5:3.5; 96:4, 95:5, 90:10; 80:20; 70:30; 60:40; 50:50; 40:60; 30:70; 20:80; and 10:90), and MeOH as mobile phase to obtain 86 primary fractions. A thin-layer chromatography (TLC; silica gel 60 F_254_, Merck Germany) profile of all the collected fractions was performed using the combinations of CHCl_3_:MeOH as mobile phase and the bands were viewed under UV lights of 254 and 366 nm wavelengths. Fractions with similar retention factor (Rf) of bands were mixed and finally grouped into 24 fractions (Fraction 1–Fraction 24). The percent yield of all the fractions was calculated and screened for *in vitro* wound-healing activity through scratch wound assay against L929 cell lines, followed by antimicrobial activity against bacterial and fungal skin pathogens.

The bioactive fraction 9, which showed a significantly higher percentage of cell migration during scratch wound assay with potential antimicrobial activity, was further subjected to column chromatography (weight of the fraction nine taken for separation: 1.006 g) using CHCl_3_, CHCl_3_:MeOH (99:1; 98:2; 97:3; 96:4, 95:5, 90:10; 80:20; 70:30; 60:40; 50:50; 40:60; 30:70; 20:80; 10:90) and MeOH as eluents to obtain 47 sub-fractions. All the sub-fractions were collectively included into 15 groups based on their Rf values on the TLC plates (Sub-fraction 9.1– Sub-fraction 15). The percent yield of all the sub-fractions was calculated and screened for *in vitro* wound-healing and antimicrobial activities. The bioactive sub-fraction 9.13 with a highly significant percentage of cell migration and antimicrobial activities yielded a major compound. It was dissolved in MeOH with activated charcoal to obtain purified off-white, non-crystalline powder after drying.

#### 2.2.1 Structure elucidation of bioactive compound

The structure of the isolated compound was determined through various spectroscopic techniques such as Fourier transform infra red (FT-IR), Nuclear magnetic resonance (NMR), Gas chromatography-mass spectrometry (GC-MS), and Ultraviolet-visible spectroscopy (UV-Vis).

IR spectra of the isolated compound were recorded on a Nicolet-5700 FT-IR spectrophotometer (KBr disc). ^1^H-NMR and ^13^C-NMR spectra were recorded on a Bruker 400 and 100 MHz spectrometer respectively, using DMSO-*d*_6_ as the eluting solvent and TMS as an internal standard. The chemical shifts were expressed as δ (ppm) and the mass spectra were recorded using the Agilent-single Quartz GC-MS instrument. UV spectra were recorded on U-3310 UV-Vis spectrophotometer and structure of the isolated compound was determined as ethyl gallate (EG) (structure elucidation results provided in Section 3.2).

### 2.3 Cell culture, cell viability/toxicity tests, and *in vitro* wound healing activity of fractions, sub-fractions, and EG through scratch wound assay

L929 mouse fibroblast cells were procured from the National Centre for Cell Science (NCCS), Pune, India. The cells were immediately thawed in a water bath at 37°C, and transferred to 25 cm^2^ T-Flask (Nunc EasYFlask) containing 7 ml complete growth medium [Dulbecco’s Modified Eagle’s Medium (DMEM), sodium bicarbonate, 10% fetal bovine serum (FBS), and 1% penicillin-streptomycin as per manufacturer’s instructions] and maintained at 37°C, 5% CO_2_ in a humidified incubator. The fresh medium was supplied a day after plating and for every 2–3 days following until cells reached 70%–80% confluence before subculture (Azis et al., 2017).

#### 2.3.1 Cell viability and toxicity assay

Cell viability and toxicity of samples (fractions, sub-fractions, and EG) was analyzed through MTT assay (3-[4,5-Dimethylthiazol-2-yl]-2,5- diphenyltetrazolium bromide) (Azis et al., 2017). Cells were seeded in a 96-well microplate (Nunc^TM^Microwell^TM^96-well microplates, ThermoFisher Scientific) at 1 × 10^4^ cells/well density, containing a complete growth medium, and incubated for 24 h. After incubation, the medium was replaced by fresh serum-free medium with various concentrations (1,000–1.95 μg/ml) of samples and the plate was incubated for a further 48 h. The cells under treatment with serum-free medium without any samples were considered as control. A serum-free medium containing 25 µL of MTT (5 mg/ml concentration) was added to each well. The medium was carefully removed from the wells followed by complete drying after 4 h of incubation. Dimethyl sulfoxide solution (DMSO, 100 μL) was put in each well with intermittent shaking of the plate for 10 min to suspend the purple-colored residual formazan crystals. Absorbance of the solution was measured spectrophotometrically with a 96-well microplate reader (Thermo Scientific Multiscan Go Version 1.00.40) at 540 nm wavelength. The percentage cell viability and toxicity (IC_50_) of all the samples were determined using ED_50_ plus v1.0 software.

#### 2.3.2 *In vitro* wound-healing activity through scratch wound assay

The cell migration activity of all the samples against L929 cells was performed through scratch wound assay as described in our previous study (Bhat et al., 2022). The cells were seeded in 24-well plates (TC plate, sterile, 24 F wells, Tarsons) containing complete medium, followed by incubation. A linear wound was created on the confluent monolayer of cells using 10 μL sterile pipette tips and the cell debris in each well was washed with phosphate buffer saline (PBS). The medium (with 3% FBS and other ingredients mentioned earlier) used consisted of either different concentrations of samples or the positive control (epidermal growth factor EGF at 0.002 μg/ml dose). The plane medium without any test samples was considered as the negative control group. Images of scratch wounds of each group were taken at the 0^th^, 24th, and 48th hours of treatment using an inverted microscope (Olympus CKX41) attached to a camera (Magcam DC5). The ×100 magnification images were photographed to evaluate the percentage migration of cells towards the scratch wound. The area between wound edges was precisely calculated using NIH ImageJ software, and the percentage wound contraction was estimated.

### 2.4 *In vitro* antimicrobial assay

The antimicrobial property of all the samples was performed using tube dilution assay against bacterial and fungal pathogens causing wound infections (Bhat et al., 2022). The standard microbial strains were obtained from the Microbial Type Culture and Collection (MTCC), Chandigarh, and the National Collection of Industrial Microorganisms (NCIM), Pune, India. *Staphylococcus aureus* (MTCC737), *Pseudomonas aeruginosa* (MTCC 1688), *Proteus vulgaris* (MTCC 1771), *Klebsiella pneumoniae* (MTCC 109), *Micrococcus luteus* (NCIM 2103), *Salmonella typhimurium* (NCIM 2501), and *Micrococcus flavus* (NCIM 2379) were the procured bacterial strains. *Trichophyton rubrum* (MTCC 296), *Microsporum canis* (MTCC 2820), *Microsporum gypseum* (MTCC 2819), *Trichophyton mentagrophytes* (MTCC 7687), *Malassezia furfur* (MTCC 1374), *Epidermophyton floccosum* (MTCC 7880), and *Candida albicans* (MTCC 183) were the fungal strains procured for antimicrobial evaluation. The media used for subculturing the strains were Sabouraud Dextrose Agar (SDA), Nutrient Agar (NA), and Potato Dextrose Agar (PDA). Peptone water was used to prepare the bacterial and fungal suspension cultures and they were incubated for 18–24 h at 37°C. The turbidity of suspension cultures was set to 1.5 × 10^6^ CFU/ml and the relative antimicrobial activity was compared with the positive standards *viz*. streptomycin (for bacterial strains), nystatin, and voriconazole (for fungal strains). The stock solutions of samples and the positive standards were dissolved in 1% DMSO to make two-fold serial dilutions ranging between 1,000–0.97 μg/ml concentrations. The tubes containing inocula were incubated for 24–48 h at 37°C and the lowest concentration of samples/standards, which inhibits the growth of microbial strains, was considered as MIC.

### 2.5 *In vitro* antioxidant activity of bioactive samples

#### 2.5.1 DPPH radical scavenging assay

DPPH activity of bioactive samples (fraction 9, sub-fraction 9.13, and EG) was measured as per the method described by Pandey et al. (2017), with few modifications. In brief, 3.5 ml DPPH solution (0.1 mM) prepared in methanol solvent was mixed with 0.5 ml standard gallic acid and samples of different concentrations (100–1.56 μg/ml) prepared in methanol. The reaction mixture was incubated at 37°C for 30 min and the absorbance of the reaction mixture was determined at 517 nm against control (DPPH stock solution) in the 96-well microplate reader.

#### 2.5.2 ABTS radical scavenging assay

ABTS radical scavenging activity of bioactive samples (fraction 9, sub-fraction 9.13, and EG) was estimated as per the method of Youn et al. (2018). The ABTS radical cation solution was prepared by mixing 88 μL potassium persulphate (140 mM) and 5 ml ABTS (7 mM) solutions. An intensely colored ABTS radical cation solution was diluted to 1:44 ratio with distilled water after 16 h of incubation. A volume of 50 µL of varying concentrations (125–1.95 μg/ml) of standard catechin and the samples dissolved in methanol were mixed with 1 ml diluted ABTS radical cation working solution. Absorbance of the reaction mixture at 734 nm was measured against control (ABTS radical cation working solution) in a 96 well microplate reader after 30 min of incubation in the dark at room temperature.

IC_50_ of samples and corresponding standards against DPPH and ABTS radicals were calculated using the ED50 plus v1.0 software program.

### 2.6 *In vivo* acute dermal toxicity studies of EG

Acute dermal toxicity of the ointment containing EG was performed as per the Organization for Economic Co-operation and Development guidelines (OECD Guideline-402) to determine the suitable doses for evaluating *in vivo* wound-healing activity (OECD, 2017). Wistar rats of either sex weighing 200–280 g (b.w.) were selected for the assessment of acute toxicity and they were acclimatized to standard room temperature (25°C ± 2°C), alternative light-dark cycle (12/12 h), and 55% relative humidity. All the experimental animals were fed with feed procured from Amrut Laboratory, Pune, India and supplied with water *ad libitum* throughout the study period. After 7 days of acclimatization, EG at 2000 mg/kg b.w. limited dose was dissolved in a simple ointment base to prepare the test dose as per Indian Pharmacopoeia (IP). The fur from the dorsal surface of the animals was removed using an electric shaver. The test was performed in control and treatment groups, each with five male and five female rats, respectively, and they were topically applied once with ointment base and EG ointment. The animals were observed for 14 days of the study period for the toxic synptoms on rat skin, viz. itching, irritation, redness, swelling, and other alterations in behavior, if any.

### 2.7 Evaluation of *in vivo* wound-healing activity of EG

Wistar rats (200–280 g) of both sexes were used to evaluate *in vivo* wound-healing activity of EG. The approval for experimental protocols and the use of animals was given by Animal Ethics Committee, S.E.T. College of Pharmacy, Sangolli Rayanna Nagar, Dharwad, India (SETCP/IAEC/2012-13/519). Animals of corresponding treatment groups were managed individually in clean separate cages for 7 days of acclimatization with standard diet and water *ad libitum*, as described earlier. Wound-healing property of EG evaluated using circular excision and linear incision wound models was as described in our previous study (Bhat et al., 2016). Different concentrations of EG (0.5% and 1%) were employed using a simple ointment base formulation with yellow soft paraffin, hard paraffin, wool fat, and cetostearyl alcohol in a 7:1:1:1 ratio as per Indian Pharmacopoeia (Biswas et al., 2013). The sample size (n) of experimental animals was statistically calculated using G* Power statistical program (Charan and Kantharia, 2013) and the number of animals in each group was finalized as *n* = 6 (either sex in each group). The animals for circular excision and linear incision wound models were categorized into the following groups:

Group 1- Ointment treated with EG 0.5% w/w.

Group 2- Ointment treated with EG 1% w/w.

Group 3- Treated with standard ointment (Positive control group; Povidone-Iodine ointment 5% w/w I.P.)

Group 4- Treated with Ointment base I.P. (Ointment base group).

Group 5- No treatment (Negative control group).

#### 2.7.1 Circular excision wound model

The experimental rats were anesthetized through the open mask method before wound creation (Bhat et al., 2022). A day prior to starting the experiment, the fur from the dorsal area of the animals was cleanly shaved with an electric shaver and disinfected with 70% ethanol. A circular excision wound of 500 mm^2^ diameter size was created with a surgical scissor on the shaved dorsal region of the rats. The wounds were topically applied with approximately 1 G of test ointments, standard Povidone-Iodine, and the ointment base to the respective group of animals once a day up to the 15th post-wounding day. The raw wound was traced every alternate day up to the end of the study (15th post-wounding day) using transparent polythene sheets sterilized with 70% ethanol and a permanent marker. The area of traced wounds was measured using millimeter scale graph sheets, and the wound progression images of all the experimental animals were taken using a Nikon D5600 camera. The percentage wound contraction in all the experimental animals was determined through the following equation:

$$\%\;wound\;contraction=\left[Wound\;area\;on\;day{\;}^{\prime }0\prime –Wound\;area\;on\;day\;\prime n\prime /\;Wound\;area\;on\;day\;\prime 0\prime \right]\times 100$$

Where, *n* = wound area on every alternate day (from 1st to 15th post wounding days). The study also includes Complete epithelialization period (CEP) in all the animals of corresponding groups.day “0”–Wound area on day “n”/Wound area on day “0” ×100.

#### 2.7.2 Linear incision wound model

A longitudinal incised wound (size: 6 cm length) was created on the shaved dorsal region of animals of corresponding groups as per standard procedure (Bhat et al., 2022). The incised wound was sutured 1 cm apart with Acos™ Sunmedix disposable sterilized skin staplers. Approximately 1 G of test ointments, standard Povidone-Iodine, and ointment base were topically applied once per day to the incised wounds of respective animals till the 15th post-wounding day. Staples were removed on the 13th post-wounding day, and the ointment application was continued up to the end of the experiment. Animals were sacrificed on the 15th post-wounding day under anesthesia and the tensile strength of each animal’s skin was determined using the Tensiometer instrument. The weight (in grams) required to break the treated incised wound was considered as tensile strength (g/mm^2^).

#### 2.7.3 Histopathology of granulation tissues

On the 15th post-wounding day, the animal skin tissue specimens of corresponding groups in the excision wound model were collected to perform histopathological studies. Skin specimens were processed and fixed in Bouin’s solution, followed by paraffin blocking. Skin sections of 5 µm thickness were taken in a microtome (Leica, RM 2145), followed by staining with hematoxylin and eosin (H&E), Masson’s trichome, and Toluidine blue stains as per standard procedures (Suntar et al., 2010). The microphotographs of sections were captured through an inbuilt analog camera (ProgRess^®^ C5-JENOPTIK) attached to Carl Zeiss Axio Imager M_2_ compound microscope and the images were analyzed at (×100 and ×400 magnifications. Quantification of different cells (neutrophils, inflammatory cells, blood vessels, myofibroblasts, mast cells, and macrophages) per unit area of images at ×100 magnification were performed using NIH ImageJ software (Hashemnia et al., 2019).

#### 2.7.4 Biochemical parameters of granulation tissues

Quantitative biochemical analysis of granulation tissues such as hydroxyproline, hexosamine, superoxide dismutase (SOD), catalase (CAT), reduced glutathione (GSH), and lipid peroxidase (LPO) were carried out as per the method described in our previous publication (Bhat et al., 2022), using skin tissue specimens of corresponding animal groups. Known quantities of dried tissues were homogenized with different solvents using a mechanical homogenizer. The homogenized tissue samples were centrifuged at 6,000 rpm for 20 min and the supernatants were stored at 4°C for further biochemical estimations.

### 2.8 Molecular docking studies of EG

Three important inflammatory drug targets viz., cyclooxygenase-2 (COX2/PTGS2), matrix metalloproteinase-9 (MMP-9), and tumor necrosis factor-alpha (TNF-α), were prioritized. Molecular docking was performed to infer the intermolecular interaction of EG with COX-2, MMP-9, and TNF-α. Three stages of molecular docking were carried out: 1) preparation of the ligand, 2) preparation of macromolecules (targets), and 3) identification of the active site and docking the ligand with the target.

(i) Preparation of the ligand: Three-dimensional (3D) structure of EG was obtained in “.sdf” format from the PubChem small molecule database (https://pubchem.ncbi.nlm.nih.gov). Using the conjugate gradients approach, the “uff” forcefield was used to minimize ligand structure. By including the gasteigers charges and polar hydrogens, the ligands were changed into “.pdbqt” format.
(ii) Preparation of macromolecules: The 3D x-ray crystallographic structure of COX-2 (5IKT) (Orlando and Malkowski, 2016), MMP-9 (4HMA) (Antoni et al., 2013), and TNF-α (2AZ5) (He et al., 2005) were retrieved from the protein data bank database of Research Collaboratory for Structural Bioinformatics (RCSB; https://www.rcsb.org/). The computed atlas of surface topography of proteins (CASTp; http://sts.bioe.uic.edu/castp/index.html?1bxw) (Tian et al., 2018) and P2Rank (https://prankweb.cz/) (Krivák and Hoksza, 2018) server were used to identify the active site of each target, and docking of the ligand was performed within the identified site.
(iii) Docking: AutoDock vina (https://autodock.scripps.edu/) was used to perform molecular docking via executing through the POAP pipeline (DasNandy et al., 2022; Patil et al., 2022). Exhaustiveness was set to 100 and BIOVIA Discovery Studio Visualizer 2019 (available for download at https://discover.3ds.com/discovery-studio-visualizer-download) was used for visualizing protein-ligand intermolecular interaction and molecules with the lowest binding energy.

#### 2.8.1 Stability of the docked complexes

All-atom molecular dynamics (MD) simulation was carried out with the docked conformations of three distinct targets for 100 ns in an explicit solvent to investigate their intermolecular interactions and structural stabilities. The execution of MD simulations with the Amber ff99SB-ildn force field was employed with the GROMACS ver. 2021.3 (https://www.gromacs.org) software package (Khanal et al., 2022; Khanal et al., 2023). Amber Tools’ xleap module (https://ambermd.org/AmberTools.php) was used to prepare the topological properties of the ligands and the whole complex. Further, an antechamber with a “bcc” charge model was used to calculate the partial charges of small molecules. The salvation of the prepared systems in a rectangular box were performed using the three-site water (TIP3P) model, with 10.0 Å boundary conditions from the protein’s boundaries in all directions. The required number of counter ions were added to neutralize the charges on the prepared systems. To establish the near-global state least energy conformations, the steepest descent and conjugate gradient energy minimization methods were used. Canonical (NVT and NPT) and isobaric (NPT) ensembles were employed for system equilibration for 1 ns. A modified Berendsen thermostat approach was adopted (300 K) to maintain the volume and temperature constant during NVT equilibration. A Parrinello-Rahman barostat was employed during NPT equilibration to keep 1 bar pressure constant. To determine van der Waals, coulomb, and long-range electrostatic interactions, the Particle Mesh Ewald approximation was utilized with 1 nm cut-off value. Similarly, to constrain the bond length, the Linear Constraint Solver approach was used. Each complex went through a 100-ns production run, with coordinates recorded every 2 fs. In-built gromacs tools were used to analyze the generated trajectories and the additional software was used for further specialized investigations, wherever appropriate.

### 2.9 Statistical analysis

The experimental parameters were performed in triplicate and the values were expressed as mean ± standard error. Statistical analyses were performed using one-way ANOVA (IBM SPSS software package, 20th version) with Tukey *post hoc* test, at significance level of *p* ≤ 0.05, *p* ≤ 0.01, and *p* ≤ 0.001.

## 3 Results

### 3.1 Percent yield of fractions, sub-fractions, and EG

Among 24 fractions collected during column chromatography, the percent yield of all the fractions was in the range of 0.30%–20.21%. The highest yield was exhibited by fraction 9 (20.21%; 202.13 mg/g), followed by fraction 12 (9.11%; 91.08 mg/g), fraction 13 (8.51%; 85.10 mg/g), and fraction 16 (7.57%; 75.72 mg/g). Among 15 sub-fractions and EG, the percent yields ranged between 1.52% and 74.29%. In comparison, EG exhibited the highest yield (74.29%; 742.86 mg/g), followed by sub-fraction 9.13 (37.81%; 378.11 mg/g), 9.15 (10.10%; 100.99 mg/g), 9.14 (8.08%; 80.84 mg/g), and 9.8 (5.48%; 54.78 mg/g) (Supplementary Table S1).

### 3.2 Structure elucidation of bioactive compound

Ethyl 3, 4, 5-trihydroxybenzoate (Ethyl gallate) IR (KBr) cm^-1^3452 (-OH), 1707 (Ester C=O); UV *λ*_max_ (EtOH): 270 nm; ^1^H-NMR (400 MHz, DMSO-*d*_*6*_, TMS) δ (ppm): 9.13 (br-s, 3H, -OH), 6.90 (s, 2H, Ar-H), 4.16 (q, *J* = 7.2 Hz, 2H, -CH_2_), 1.23 (t, *J* = 7.2 Hz, 3H, -CH_3_); ^13^C-NMR (100 MHz, DMSO-*d*_*6*_) δ (ppm): 14.21, 59.92, 108.42, 119.54, 138.28, 145.50, and 165.77; EI-MS *m/z*198.

**FIGURE 2:**
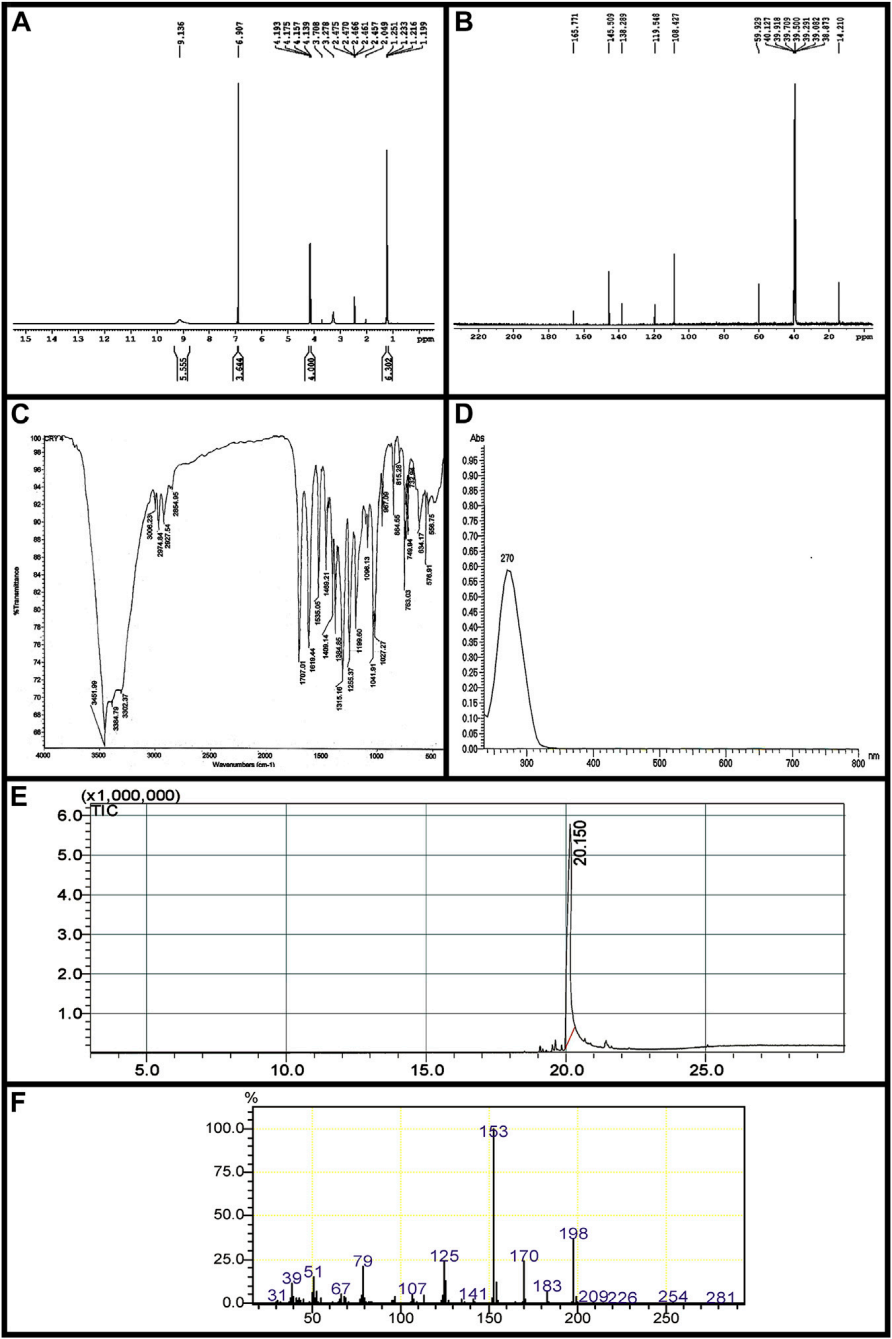
Characterization of EG. **(A)** ^1^H-NMRspectrum, **(B)** ^13^C-NMR spectrum, **(C)** FT-IR **(D)** UV- Vis spectra, **(E)** GC chromatogram, **(F)** EI-MS.

**FIGURE 3:**
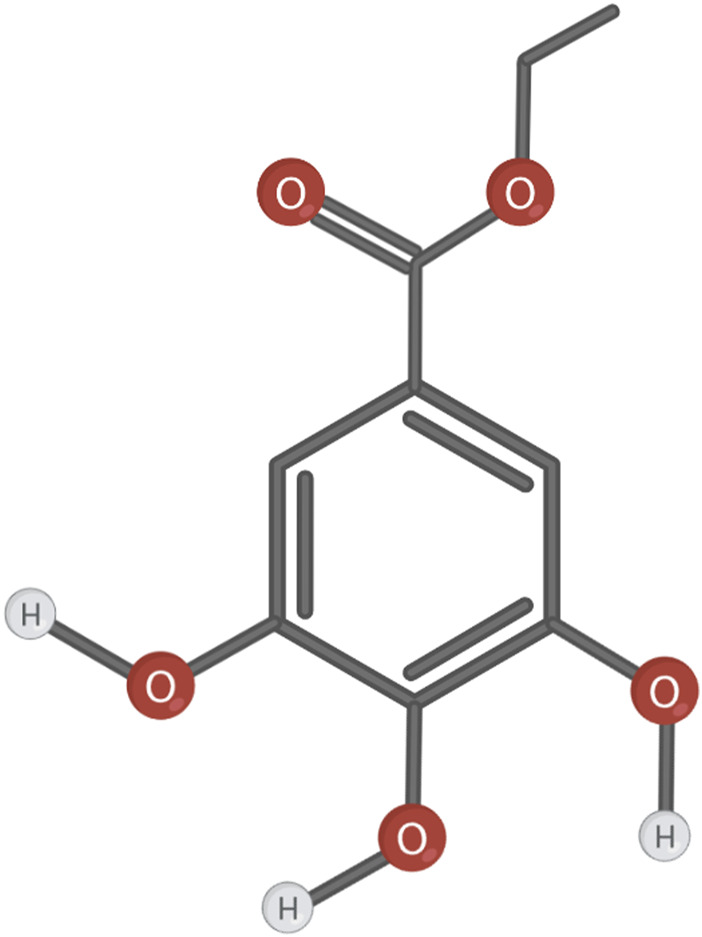
Chemical structure of the compound EG.

IR spectra showed–OH stretching at 3,452 cm^−1^; a band at 1707 cm^−1^ exhibits typical ester stretching. In ^1^H-NMR, the ethoxy protons resonated in triplet-quartet pattern at 1.23 ppm and 4.16 ppm with *J*-value of 7.2 Hz. The two symmetrical aromatic protons resonated at 6.90 ppm as a singlet. A broad singlet observed at 9.13 ppm corresponds to three hydroxyl protons. Further, in ^13^C-NMR, the methyl carbon resonated at 14.21 ppm. A signal at 59.92 ppm corresponds to methylene carbon. The carbonyl carbon of the ester was observed in the most downfield region at 165.77 ppm. The signal at 119 ppm is attributed to the methine carbon attached to the carbonyl carbon. The methine carbon *ortho* to hydroxyl group resonated at 108.42 ppm, whereas the two carbons *meta* and *para* to the ester functionality were observed at 145.50 and 138.28 ppm, respectively. The molecular ion peak at *m/z* 198 confirms the compound ethyl gallate (Figure 2, Figure 3).

### 3.3 Cell viability and toxicity of fractions, sub-fractions, and EG

L929 cells were exposed to different concentrations (1,000–1.95 μg/ml) of fractions, sub-fractions, and EG for 24 h and the cytotoxicity was analyzed through MTT assay. The percentage viability of L929 cells showed a gradual increase with increasing concentration of all the samples (fractions, sub-fractions, and EG) up to certain limits (1.95–15.62 μg/ml in fractions and sub-fractions, 0.975–7.81 μg/ml in EG). It later decreased rapidly with increased concentrations. It was found that all the fractions (1–24) and sub-fractions (9.1–9.15) showed ≥100% cell viability at 15.62–7.81 μg/ml concentrations (Supplementary Figures S1A, B). However, none of them exhibited 100% cell viability when they were treated with more than 15.62 μg/ml concentration of fractions, sub-fractions, and EG. In the case of EG, more than 100% cell viability was observed only when the cells were treated with 7.81, 3.90, 1.95, and 0.975 μg/ml concentrations (Supplementary Figure S1C).

Among all the fractions, the highest percentage of cell viability was achieved by fraction 2 (108.27 ± 0.16%), followed by fraction 9 (106.21 ± 0.77%), fraction 6 (104.48 ± 1.10%), and fraction 4 (103.74 ± 0.96%) at 15.62 μg/ml concentration (Supplementary Figure S1A). While among the sub-fractions, it was 114.54 ± 1.44%, 112.03 ± 0.90%, 110.45 ± 1.61%, and 109.24 ± 0.41% for sub-fraction 9.13, sub-fraction 9.3, sub-fraction 9.1, and sub-fraction 9.2 respectively at 7.81 μg/ml concentration (Supplementary Figure S1B). The isolated compound EG showed a higher percentage of cell viabilities (122.19 ± 2.85%, 117.40 ± 3.82%, and 103.49 ± 3.54%) at 1.95, 3.90, and 0.975 μg/ml concentrations respectively (Supplementary Figure S1C).

The IC_50_ of cell toxicity was found to be 323.75 ± 2.19, 312.01 ± 2.14, 307.51 ± 4.60, and 302.87 ± 6.49 μg/ml for fraction 6, fraction 15, fraction 9, and fraction 7 respectively (Supplementary Figure S1D). Among sub-fractions, it was 188.48 ± 2.19, 181.80 ± 1.78, 177.58 ± 2.32, and 167.61 ± 1.59 μg/ml for sub-fraction 9.7, sub-fraction 9.9, sub-fraction 9.2, and sub-fraction 9.10 respectively (Supplementary Figure S1E). While in the case of EG, the IC_50_ was determined at 104.08 ± 1.19 μg/ml (Supplementary Figure S1F).

### 3.4 *In vitro* wound-healing activity of fractions, sub-fractions, and EG through scratch wound assay

Based on cell viability studies, the optimum concentrations that showed more than 100% cell viability in all 24 fractions (15.62 μg/ml), 15 sub-fractions (7.81 μg/ml), and EG (3.90 μg/ml and 1.95 μg/ml) were chosen to carry out *in vitro* scratch wound assay. The effect of test samples and positive and negative controls on the migration rate of L929 cells were measured quantitatively and critically analyzed at time intervals of 24 and 48 h respectively. Among all 24 fractions, a significantly higher percentage of cell migration was observed in the cells treated with fraction 9 (92.75 ± 0.09%) compared to the negative control group (60.55 ± 1.14%) at the incubation period of the 48th hour. In the positive control group, the migration rate was increased to 98.44 ± 0.36% at the 48th hour of the incubation period, which was comparable to the effect of fraction 9 (Table 1). Therefore, fraction nine was selected for further separation process in the next phase of column chromatography.

**TABLE 1:** Percentage cell migration in L929 cells treated with 24 fractions.

Samples	Concentration	Cell migration (%) Mean ± SE	
		24th hour	48th hour
Fraction 1	15.62 μg/ml	38.75 ± 0.15^a^	65.27 ± 0.10^a^
Fraction 2	15.62 μg/ml	58.16 ± 0.14^b^	66.99 ± 0.18^a^
Fraction 3	15.62 μg/ml	40.26 ± 0.11^c^	71.56 ± 0.33^c^
Fraction 4	15.62 μg/ml	18.77 ± 0.72^e^	73.60 ± 0.35^c^
Fraction 5	15.62 μg/ml	19.71 ± 0.21^e^	61.22 ± 0.19^a^
Fraction 6	15.62 μg/ml	16.23 ± 0.23^e^	69.07 ± 1.97^a^
Fraction 7	15.62 μg/ml	20.22 ± 0.16^e^	61.27 ± 0.14^a^
Fraction 8	15.62 μg/ml	14.01 ± 0.33^e^	69.27 ± 0.19^a^
Fraction 9	15.62 μg/ml	63.40 ± 0.24^f^	**92.75 ± 0.09** ^**e**^
Fraction 10	15.62 μg/ml	21.05 ± 0.14^e^	65.00 ± 0.12^a^
Fraction 11	15.62 μg/ml	14.72 ± 0.20^e^	57.55 ± 0.16^b^
Fraction 12	15.62 μg/ml	57.63 ± 0.24^b^	81.86 ± 0.11^days^
Fraction 13	15.62 μg/ml	55.16 ± 0.13^b^	85.72 ± 0.23^days^
Fraction 14	15.62 μg/ml	53.63 ± 0.04^b^	74.60 ± 0.06^c^
Fraction 15	15.62 μg/ml	34.11 ± 3.20^a^	74.11 ± 1.80^c^
Fraction 16	15.62 μg/ml	53.05 ± 2.70^b^	74.89 ± 2.24^c^
Fraction 17	15.62 μg/ml	44.03 ± 1.2^cd^	63.79 ± 2.46^a^
Fraction 18	15.62 μg/ml	34.15 ± 0.10^a^	60.95 ± 0.10^a^
Fraction 19	15.62 μg/ml	27.78 ± 0.16^a^	71.63 ± 0.69^c^
Fraction 20	15.62 μg/ml	36.97 ± 0.05^a^	69.49 ± 0.11^a^
Fraction 21	15.62 μg/ml	37.37 ± 0.13^a^	70.08 ± 0.16^a^
Fraction 22	15.62 μg/ml	34.45 ± 0.14^a^	68.09 ± 0.30^a^
Fraction 23	15.62 μg/ml	30.71 ± 0.18^a^	65.46 ± 0.08^a^
Fraction 24	15.62 μg/ml	47.42 ± 0.09^cd^	64.04 ± 0.07^a^
PC (EGF)	0.002 μg/ml	89.61 ± 0.10^g^	**98.44 ± 0.36** ^**f**^
NC	-	31.13 ± 1.73^a^	60.55 ± 1.14^a^

SE, standard error; PC, positive control; EGF, epidermal growth factor; NC, Negative control. Different letters (^a-z^) in each column indicate significant differences among the variables by Tukey Post hoc analysis at *p* ≤ 0.05. The bold values provided in the Table represents significantly higher results compared to other groups.

**FIGURE 4:**
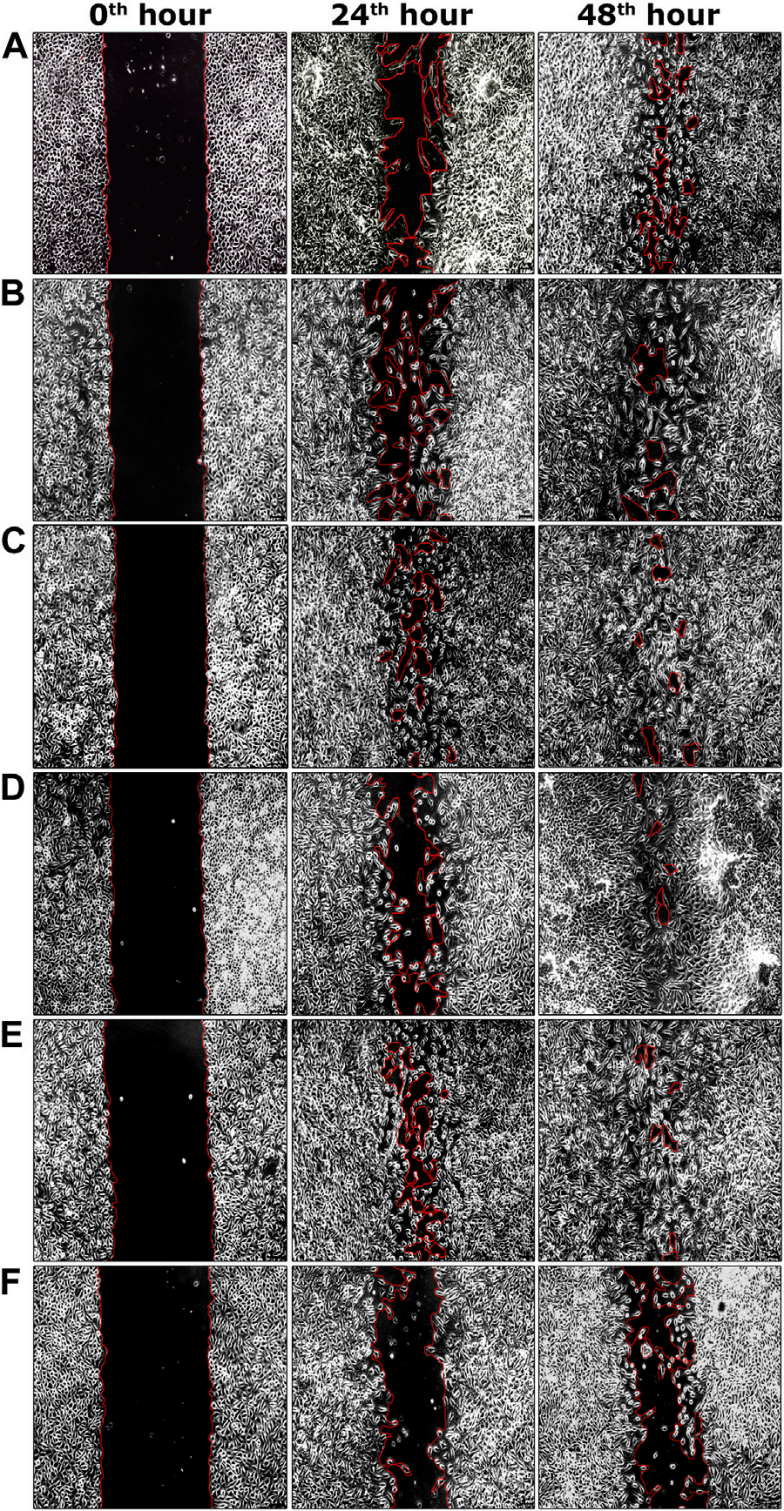
Representative images of percentage cell migration of L929 cells in scratch wound assay. **(A)** Fraction nine at 15.62 μg/ml concentration. **(B)** Sub-fraction 9.13 at 7.81 μg/ml concentration. **(C)** EG at 1.95 μg/ml concentration. **(D)** EG at 3.90 μg/ml concentration. **(E)** Positive control (EGF) at 0.002 μg/ml concentration. **(F)** Negative control. Scale bars represent 5 µm size at the original magnification of ×100.

During the second phase of the chromatographic separation process, 15 sub-fractions (sub-fractions 9.1–9.15) were obtained and were evaluated for scratch wound assay. Among all sub-fractions, sub-fraction 9.13 exhibited a significantly higher percentage of cell migration (94.62 ± 0.17%) at the 48th hour of the incubation period compared to the negative control group (60.55 ± 1.14%) (Table 2). The highly active sub-fraction 9.13 was further purified to obtain the compound EG and it was evaluated for scratch wound assay at 1.95 and 3.90 μg/ml concentrations. Significantly increased cell migration rate (97.98 ± 0.46%) was attained by the cells treated with EG at 3.90 μg/ml concentration, which was significantly similar to the positive control (98.44 ± 0.36%) at the 48th hour of incubation (Table 2). The images of percentage cell migration in all the treatment groups are given in Figure 4.

**TABLE 2:** Percentage cell migration in L929 cells treated with 15 sub-fractions and EG.

Samples	Concentration	Cell migration (%) Mean ± SE	
		24th hour	48th hour
Sub fraction 9.1	7.81 μg/ml	50.16 ± 0.20^a^	64.34 ± 0.26^a^
Sub fraction 9.2	7.81 μg/ml	41.96 ± 0.25^b^	60.60 ± 0.17^a^
Sub fraction 9.3	7.81 μg/ml	52.47 ± 0.12^a^	67.61 ± 0.22^a^
Sub fraction 9.4	7.81 μg/ml	41.94 ± 0.13^b^	62.61 ± 0.06^a^
Sub fraction 9.5	7.81 μg/ml	43.46 ± 0.19^b^	62.64 ± 0.14^a^
Sub fraction 9.6	7.81 μg/ml	27.77 ± 1.44^c^	75.89 ± 1.72^b^
Sub fraction 9.7	7.81 μg/ml	23.49 ± 0.07^days^	69.95 ± 2.00^b^
Sub fraction 9.8	7.81 μg/ml	39.48 ± 0.12^b^	66.94 ± 0.21^a^
Sub fraction 9.9	7.81 μg/ml	34.13 ± 0.06^e^	73.25 ± 0.39^b^
Sub fraction 9.10	7.81 μg/ml	40.52 ± 0.31^b^	66.66 ± 0.48^a^
Sub fraction 9.11	7.81 μg/ml	37.06 ± 0.24^e^	74.03 ± 1.39^b^
Sub fraction 9.12	7.81 μg/ml	40.56 ± 0.06^b^	67.43 ± 0.18^a^
Sub fraction 9.13	7.81 μg/ml	57.81 ± 0.15^f^	**94.62 ± 0.17** ^**c**^
Sub fraction 9.14	7.81 μg/ml	40.38 ± 0.08^b^	67.01 ± 0.23^a^
Sub fraction 9.15	7.81 μg/ml	39.82 ± 0.40^b^	66.94 ± 0.27^a^
EG	1.95 μg/ml	57.58 ± 1.18^f^	92.09 ± 0.19^c^
EG	3.90 μg/ml	48.39 ± 0.83^a^	**97.98 ± 0.46** ^**c**^
PC (EGF)	0.002 μg/ml	89.61 ± 0.10^g^	**98.44 ± 0.36** ^**c**^
NC	-	31.13 ± 1.73^c^	60.55 ± 1.14^a^

SE, standard error; PC, positive control; EGF, epidermal growth factor; NC, Negative control. Different letters (^a-z^) in each column indicate significant differences among the variables by Tukey Post hoc analysis at *p* ≤ 0.05. The bold values provided in the Table represents significantly higher results compared to other groups.

### 3.5 *In vitro* antimicrobial activity of fractions, sub-fractions, and EG

Among all the samples tested, only fraction 9, fraction 12–fraction 17, fraction 19, sub-fractions 9.12–9.15, and EG exhibited considerably effective antibacterial activity with MIC<1,000 μg/ml (Table 3). Other than these samples, the other fractions and sub-fractions failed to reveal effective antibacterial activity and exhibited the MIC>1,000 μg/ml against all the bacterial strains. Among the tested fractions, fraction nine was effective against *P. aeruginosa*, *M. luteus*, and *M. flavus* with the least MICs (7.81 μg/ml each), followed by *P. vulgaris* and *S. aureus* with 15.62 μg/ml MICs respectively. In the case of sub-fractions, the sub-fraction 9.13 was highly active with the least MIC (3.90 μg/ml) against *P. aeruginosa*, followed by *P. vulgaris*, *M. luteus*, and *M. flavus* (7.81 μg/ml each). The isolated compound EG was found efficient against *P. aeruginosa*, *S. aureus*, and *M. luteus* with the least MICs (3.90 μg/ml each), followed by *P. vulgaris* and *M. flavus* (7.81 μg/ml each), which were comparable to the standard streptomycin.

**TABLE 3:** Antibacterial and antifungal activity of fractions, sub-fractions, and EG through tube dilution method (MIC).

Fractions/Sub-fractions/EG/Standards	Bacterial strains MIC (µg/ml)						
	*Pa*	*St*	*Pv*	*Sa*	*Kp*	*Ml*	*Mf*
Fraction 9	7.81	31.25	15.62	15.62	31.25	7.81	7.81
Fraction 12	62.5	125	62.5	62.5	125	62.5	125
Fraction 13	62.5	250	125	62.5	250	125	250
Fraction 14	125	500	125	500	>1,000	125	125
Fraction 15	500	500	250	500	>1,000	250	500
Fraction 16	125	250	250	>1,000	500	250	500
Sub-fraction 9.12	125	62.5	500	125	250	125	250
Sub-fraction 9.13	3.9	15.62	7.81	15.62	62.5	7.81	7.81
Sub-fraction 9.14	250	62.5	62.5	125	125	125	250
Sub-fraction 9.15	500	125	125	62.5	125	125	250
EG	3.9	15.62	7.81	3.9	15.62	3.9	7.81
Streptomycin (Std)	3.9	7.81	3.9	7.81	3.9	1.95	1.95
**Fractions/Sub-fractions/EG/Standards**	**Fungal strains MIC (µg/ml)**						
	***Tr***	***Tm***	***Mc***	***Mg***	***Ef***	***Mfu***	***Ca***
Fraction 9	7.81	62.5	250	31.25	500	15.62	31.25
Fraction 12	500	500	>1,000	>1,000	>1,000	62.5	125
Fraction 13	7.81	62.5	>1,000	31.25	>1,000	125	62.5
Fraction 16	15.62	62.5	500	62.5	>1,000	125	62.5
Sub-fraction 9.13	15.62	31.25	250	7.81	125	15.62	7.81
EG	7.81	15.62	250	3.9	62.5	7.81	7.81
Nystatin (Std)	3.9	7.81	7.81	1.95	7.81	1.95	1.95
Voriconazole (Std)	1.95	0.975	0.975	3.9	3.9	0.975	1.95

Std, Standard; Pa, *Pseudomonas aeruginosa*; St, *Salmonella typhimurium*; Pv, *Proteus vulgaris*; Sa, *Staphylococcus aureus*; Kp, *Klebsiella pneumoniae*; Ml, *Micrococcus luteus*; Mf, *Micrococcus flavus*; Tr, Trichophyton rubrum; Tm, Trichophyton mentagrophytes; Mc, Microsporum canis; Mg, Microsporum gypseum; Ef, Epidermophyton floccosum; Mfu, Malassezia furfur; Ca, *Candida albicans*.

Similarly, fraction 9, fraction 12, fraction 13, fraction 16, sub-fraction 9.13, and EG exhibited considerably effective antifungal activity with MIC<1,000 μg/ml (Table 3). The other fractions and sub-fractions failed to reveal effective antifungal activity and exhibited MIC>1,000 μg/ml against all the fungal strains. Among the fractions tested, fraction nine was found to be effective with the least MIC against *T. rubrum* (7.81 μg/ml), followed by *M. furfur* (15.62 μg/ml), *M. gypseum*, and *C. albicans* (31.25 μg/ml each). Sub-fraction 9.13 effectively inhibited the growth of *M. gypseum* and *C. albicans* with the lowest MICs (7.81 μg/ml each). Similar results were also observed in EG against *M. gypseum* (3.90 μg/ml), *T. rubrum*, *M. furfur*, and *C. albicans* with 7.81 μg/ml MICs each, which were comparable to Nystatin and Voriconazole standards.

### 3.6 *In vitro* antioxidant activity

Antioxidant activity of the active samples, such as fraction 9, sub-fraction 9.13, and EG, were carried out using DPPH and ABTS scavenging assays. The percentage scavenging activity of standard gallic acid and the samples were increased in a dose-dependent mode and ranged from 28.38 ± 0.47% to 85.87 ± 0.36% (fraction 9), 28.38 ± 0.47% to 91.22 ± 0.47% (sub-fraction 9.13), 23.25 ± 0.79% to 98.42 ± 0.20% (EG), and 27.20 ± 0.34% to 98.87 ± 0.20% (standard gallic acid) at 1.95 and 125 μg/g concentrations respectively (Table 4). Interestingly, the gallic acid and EG showed significantly similar IC_50_ results with 8.33 ± 0.41 and 8.83 ± 0.71 μg/g respectively, which revealed the potential antioxidant activity of EG (Supplementary Table S2).

**TABLE 4:** Antioxidant activity (percentage scavenging) of fraction 9, sub fraction 9.13, EG, and reference standards.

Concentration of samples (μg/ml)	Scavenging activity (%)							
	DPPH				ABTS			
	Fraction 9	Sub fraction 9.13	EG	Standard gallic acid	Fraction 9	Sub fraction 9.13	EG	Standard catechin
125	85.87 ± 0.36	91.22 ± 0.47	98.42 ± 0.20	98.87 ± 0.20	80.12 ± 0.42	89.54 ± 0.55	98.68 ± 0.29	98.68 ± 0.57
62.5	69.26 ± 0.28	81.14 ± 0.42	91.61 ± 0.26	92.51 ± 0.73	55.79 ± 0.87	77.98 ± 0.76	86.78 ± 0.19	87.11 ± 0.75
31.25	50.79 ± 0.46	72.19 ± 0.19	79.96 ± 0.40	80.74 ± 0.25	49.83 ± 0.72	59.75 ± 0.54	77.77 ± 0.57	78.26 ± 0.22
15.62	43.41 ± 0.36	50.56 ± 0.15	67.23 ± 0.41	71.90 ± 0.42	37.98 ± 3.43	50.66 ± 0.24	56.82 ± 0.24	60.16 ± 0.72
7.81	38.62 ± 0.41	43.19 ± 0.55	46.45 ± 0.40	42.00 ± 0.41	34.51 ± 2.44	48.39 ± 0.49	48.14 ± 0.32	48.39 ± 0.58
3.9	33.73 ± 0.62	33.56 ± 0.47	36.65 ± 0.45	34.35 ± 0.09	28.22 ± 0.76	29.63 ± 0.42	33.47 ± 0.11	34.42 ± 0.59
1.95	28.38 ± 0.47	28.38 ± 0.47	23.25 ± 0.79	27.20 ± 0.34	20.74 ± 0.70	20.37 ± 0.27	19.42 ± 0.31	17.19 ± 0.82

Similarly, ABTS radicals were scavenged by standard catechin and the active samples in a dose-dependent manner. Standard catechin exhibited 98.68 ± 0.57% scavenging at 125 μg/g concentration and the samples were also active with the scavenging percentages of 80.12 ± 0.42% (fraction 9), 89.54 ± 0.55% (sub-fraction 9.13), and 98.68 ± 0.29% (EG) at the same concentration (Table 4). From the results, it was clear that the scavenging activity of both EG and standard catechin were close to each other and were supported by IC_50_ values with significantly similar results (15.99 ± 0.33 and 15.12 ± 0.42 μg/g respectively) (Supplementary Table S2).

### 3.7 *In vivo* acute dermal toxicity of EG

Acute dermal toxicity of isolated compound EG was evaluated and no indications of any toxic symptoms on experimental animals were detected at 2000 mg/kg limited dose. Toxic signs *viz*. changes in mucous membranes, fur, locomotory organs, eyes, or on other body parts of the animals were not reported during the study period. Any adverse effects of EG on animal skin *viz*. itching, swelling, redness, irritation, other behavioral patterns, and mortality of the animals were also not noticed during the toxicity study.

### 3.8 *In vivo* wound-healing activity of EG

#### 3.8.1 Circular excision wound model

**FIGURE 5:**
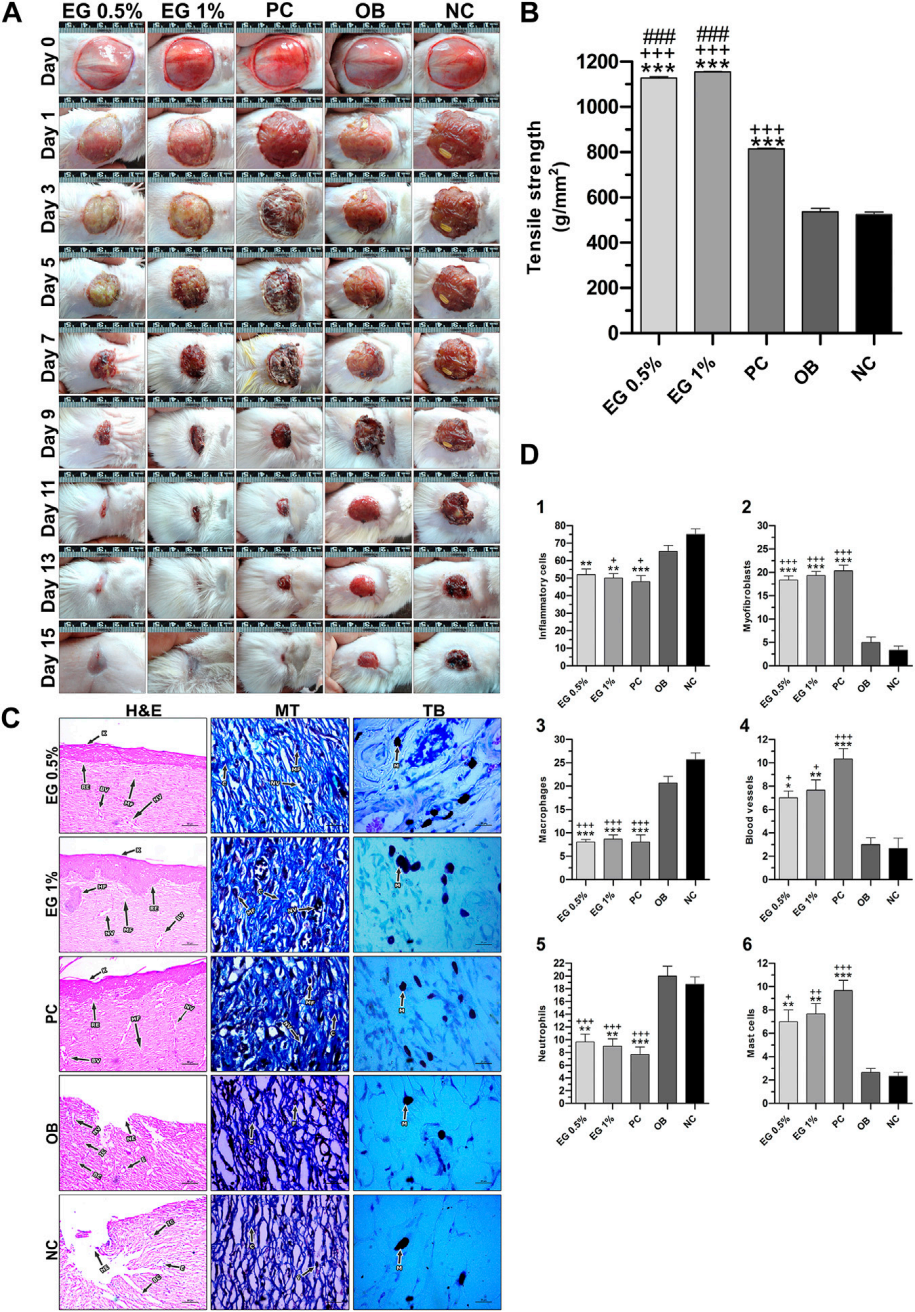
*In vivo* wound-healing parameters of EG. **(A)** Wound contraction results on different post-wounding days. **(B)** Effect of test ointments on incision wound model. **(C)** Histopathological view of excision wound model. MT, Masson’s trichome; TB, Toluidine blue. In H&E-stained sections, the magnification was set at ×100 with 200 µm scale bars. The magnification was set at ×400 with 20 µm scale bars for MT and TB-stained sections. Arrows representing the cell components are BC, Blood clot; BV, Blood vessel; C, Collagen; E, Edema; F, Fibroblast; HF, Hair follicle; IC, Inflammatory cell; K, Keratinization; MA, Macrophage; MC, Mast cell; N, Neutrophil; MF, Myofibroblast; NE, Necrosis; NV,Neovascularization; RE, Re-epithelization. **(D)** Quantification of cell constituents (1) Inflammatory cells, (2) Myofibroblasts, (3) Macrophages, (4) Blood vessels, (5) Neutrophils, (6) Mast cells. ‘*’ represents the significance levels at *p ≤ 0.05, **p ≤ 0.01, or ***p ≤ 0.001 among corresponding treatment groups and negative control group; ‘+’ represents the significance levels at + *p* ≤ 0.05, ++*p* ≤ 0.01, or +++*p* ≤ 0.001 among corresponding treatment groups and ointment base group; ‘#’ represents the significance levels at #*p* ≤ 0.05, ##*p* ≤ 0.01, or ###*p* ≤ 0.001 among corresponding treatment groups and positive control group with Tukey *post hoc* test. PC, Positive control group; OB, Ointment base group; NC, Negative control group.

In the circular excision wound model, 1% EG ointment-treated group started showing a significantly higher rate of wound contraction (*p* ≤ 0.01 and *p* ≤ 0.001) compared to the ointment base, the negative control, and even the positive control groups from the third day up to the 11th post-wounding day (Table 5). Thereafter, a significantly elevated percentage of wound closure was observed from the 13th to 15th day of post-wounding, when compared to the ointment base and the negative control groups. Whereas in the 0.5% EG ointment-treated group, a significantly higher wound-closure rate (*p* ≤ 0.05, *p* ≤ 0.01 and *p* ≤ 0.001) was observed from the 5th to 11th post-wounding day compared to all three treatment groups (ointment base, negative control and positive control) and later on it was limited to only ointment base and negative control groups. Interestingly, on the 15th post-wounding day, 0.5% and 1% EG ointment-treated groups showed relatively higher percentages of wound contraction (98.10 ± 0.20% and 98.72 ± 0.41% respectively) when compared to the positive control group (96.84 ± 1.27%). Effective wound-healing activity of 1% EG ointment, positive control, and 0.5% EG ointment could be noticed by significantly less CEP in all three treatment groups (15.33 ± 0.33, 15.67 ± 0.67, and 16.00 ± 0.58 days respectively) in comparison with the ointment base and negative control (21.67 ± 0.33 and 22.00 ± 0.58 days) groups respectively. The photographic images of wound contraction in different treatment groups of excision wound models between the 0^th^ to15^th^ post-wounding days are given in Figure 5A.

**TABLE 5:** The effect of test ointments on percentage contraction of circular excision wound.

Percentage wound contraction (Mean ± S.E)					
Days	EG0.5%	EG1%	Positive control	Ointment base	Negative control
Day 1	1.87 ± 1.45	3.91 ± 2.36	2.67 ± 1.31	4.09 ± 3.51	0.24 ± 0.03
Day 3	24.10 ± 1.88^b***,c*^	30.58 ± 2.62^a**,b***,c***^	10.05 ± 2.66	13.49 ± 3.82	4.34 ± 3.63
Day 5	39.71 ± 4.08^a**,b*,c**^	38.08 ± 2.66^a**,b*,c**^	13.72 ± 4.20	17.89 ± 3.66	20.33 ± 5.70
Day 7	68.55 ± 1.88^a***,b***,c***^	66.89 ± 2.66^a***,b***,c***^	28.23 ± 5.20	24.00 ± 5.26	27.07 ± 8.54
Day 9	80.93 ± 0.91^a***,b***,c**^	83.59 ± 1.08^a***,b***,c**^	48.66 ± 10.57	36.79 ± 3.30	36.10 ± 11.00
Day 11	94.19 ± 0.54^a***,b**,c*^	94.09 ± 1.43^a***,b**,c*^	74.38 ± 3.94	59.26 ± 7.11	51.32 ± 5.11
Day 13	96.20 ± 0.45^a***,b***^	97.01 ± 0.79^a***,b***^	90.84 ± 3.74^a**,b**^	72.36 ± 3.00	64.32 ± 2.08
Day 15	98.10 ± 0.20^a**,b***^	98.72 ± 0.41^a**,b***^	96.84 ± 1.27^b*^	80.05 ± 3.12	70.34 ± 4.81
CEP in days	16.00 ± 0.58^a***,b***^	15.33 ± 0.33^a***,b***^	15.67 ± 0.67^a***,b***^	21.67 ± 0.33	22.00 ± 0.58

S.E, standard error; CEP, Complete epithelialization period. Significance level at *p ≤ 0.05; **p ≤ 0.01; ***p ≤ 0.001 were noted between the variables of test groups at different days of post-wounding with Tukey *post hoc* test. Different letters (^a,b,c^) in each column exhibit significant difference against ointment base, negative control, and positive control groups respectively.

#### 3.8.2 Linear incision wound model

Similarly, the results of the incision wound demonstrated the wound-healing efficacy of 0.5% and 1% EG ointments with significantly higher tensile strength (1,127.42 ± 6.24 and 1,154.60 ± 1.42 g/mm^2^ respectively at *p* ≤ 0.001) compared to a positive control (814.02 ± 3.59 g/mm^2^), ointment base (536.78 ± 14.78 g/mm^2^), and negative control (524.30 ± 11.34 g/mm^2^) groups (Figure 5B).

#### 3.8.3 Histopathology of granulation tissues

Histopathological examination of granulation tissues of animals of corresponding groups in the excision wound model was carried out on the 15th day post-wounding (Figure 5C). Through H&E-stained images, greater regeneration of granulation tissues was observed in 0.5% and 1% EG treated groups, followed by the positive control group. Complete re-epithelialization was observed in these treatment groups with the formation of the *epidermis* containing 2-3 layers of epithelial cells, stratified keratinization over the epidermal layer and formation of hair follicles. Differential cell count results in positive control and 1% EG groups illustrated a greater number of newly formed blood vessels (10.33 ± 0.88 and 7.67 ± 0.88), thickly populated myofibroblasts (20.33 ± 1.20 and 19.33 ± 0.88), and mast cells (9.67 ± 0.88 and 7.67 ± 0.88) respectively. Significant reduction in the number of inflammatory cells, neutrophils, and macrophages established the effectiveness of 1% EG and positive control groups in the successful regeneration and remodeling of wounded skin tissues (Figure 5D). It was clear from the Masson’s trichome stained sections, with intense blue color staining of well-organized mature collagen fibers in the granulation tissues. In contrast, ointment base and negative control groups showed an incomplete healing process with a lack of epidermal layer formation, edema, blood clots, infiltration of exudates, and fibrinoid necrosis. Increased cell counts of inflammatory cells, neutrophils, and macrophages in the granulation tissues indicated the poor matrix organization in the ointment base and negative control groups.

#### 3.8.4 Biochemical parameters of granulation tissues

**FIGURE 6:**
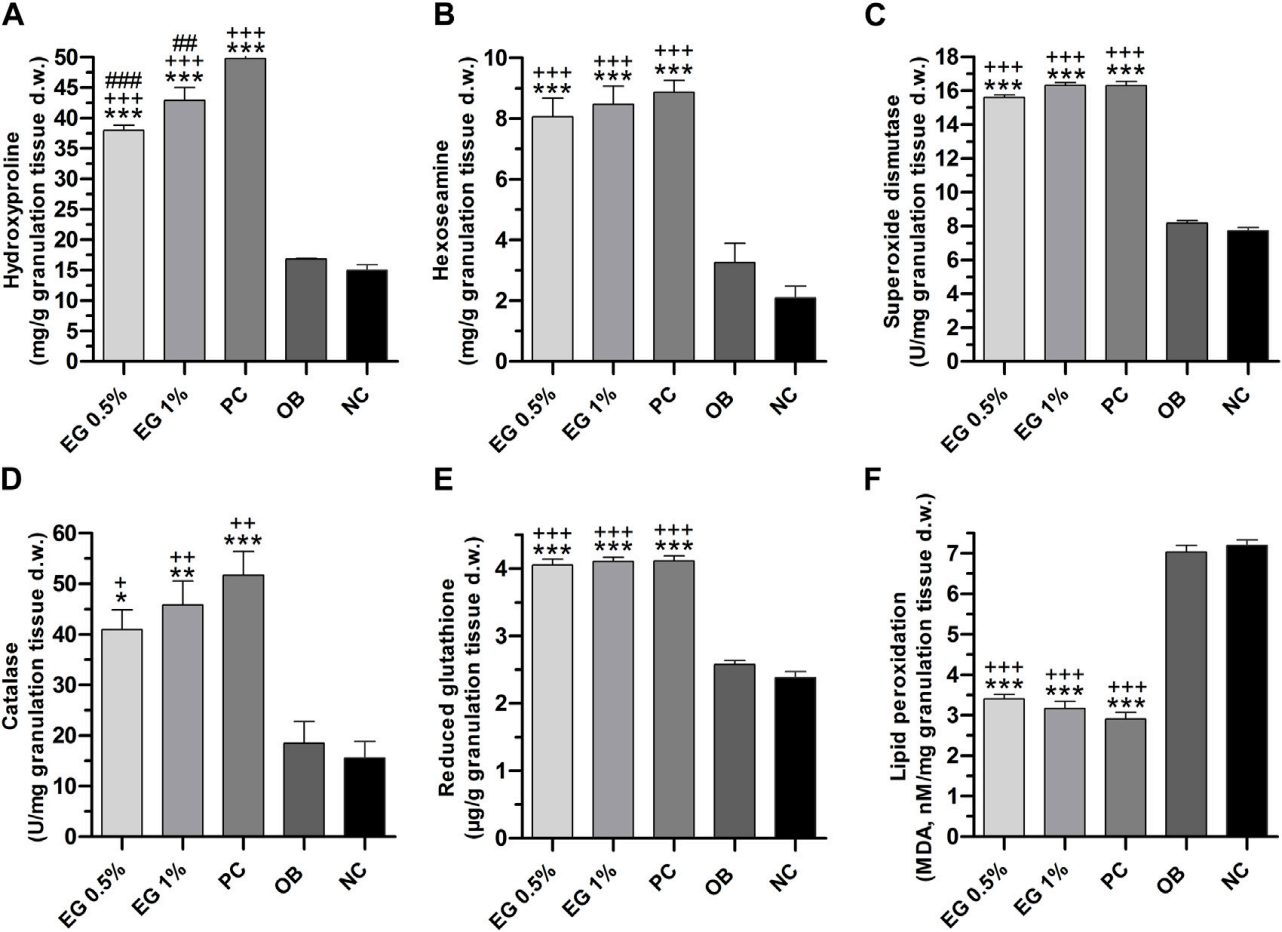
Biochemical parameters of granulation tissues in different treatment groups. **(A)** Hydroxyproline, **(B)** Hexosamine, **(C)** SOD, **(D)** Catalase, **(E)** GSH, **(F)** LPO. ‘*’ represents the significance levels at *p ≤ 0.05, **p ≤ 0.01, or ***p ≤ 0.001 among corresponding treatment groups and negative control group; ‘+’ represents the significance levels at + *p* ≤ 0.05, ++*p* ≤ 0.01 or +++*p* ≤ 0.001 among corresponding treatment groups and ointment base group; ‘#’ represents the significance levels at #*p* ≤ 0.05, ##*p* ≤ 0.01, or ###*p* ≤ 0.001 among corresponding treatment groups and positive control group with Tukey *post hoc* test. PC, Positive control group; OB, Ointment base group; NC, Negative control group.

Biochemical parameters of connective tissue elements (collagen fibers) such as hydroxyproline and hexosamine in the granulation tissues of all the treatment groups were measured on the 15th post-wound day. A significantly higher quantity (*p* ≤ 0.001) of hydroxyproline content was achieved by positive control (49.82 ± 0.44 mg/g), followed by 1% EG (42.92 ± 2.12 mg/g) and 0.5% EG (37.97 ± 0.88 mg/g) ointment treated groups, compared to ointment base (16.87 ± 0.07 mg/g) and negative control (14.97 ± 0.92 mg/g) groups (Figure 6A). Whereas hexosamine contents in the positive control showed 1% and 0.5% EG ointment treated groups were significantly high (8.87 ± 0.39, 8.47 ± 0.60 and 8.07 ± 0.61 mg/g respectively) compared to ointment base (3.27 ± 0.63 mg/g) and negative control (2.10 ± 0.38 mg/g) groups (Figure 6B).

The quantity of enzymatic antioxidant elements such as SOD and CAT enzymes in the granulation tissues were significantly increased (*p* ≤ 0.001) in 1% EG (16.31 ± 0.15 and 45.83 ± 4.76 U/mg respectively), positive control (16.30 ± 0.24 and 51.72 ± 4.65 U/mg), and 0.5% EG (15.60 ± 0.15 and 40.97 ± 3.39 U/mg) ointment treated groups, in comparison with ointment base and negative control groups (Figures 6C, D).

Similarly, a significantly increased quantity of a non-enzymatic antioxidant element i.e. GSH was observed in the positive control (4.11 ± 0.07 μg/g), 1% EG (4.10 ± 0.06 μg/g), and 0.5% EG (4.05 ± 0.09 μg/g) ointment-treated groups, in comparison with ointment base and negative control groups, with 2.58 ± 0.06 and 2.38 ± 0.09 μg/g respectively (Figure 6E).

A significant downregulation of LPO, the oxidative stress-mediated indicator, was reported in the positive control and 1% and 0.5% EG ointment-treated groups with 2.91 ± 0.16, 3.16 ± 0.18, and 3.40 ± 0.11 nM/mg compared to ointment base (7.03 ± 0.16 nM/mg) and negative control (7.20 ± 0.14 nM/mg) groups (Figure 6F). Downregulation of LPO content in the granulation tissue treated with positive control and 1% EG ointment-treated groups clearly indicates their potentiality in preventing acute inflammation and oxidative tissue damage during the process of wound healing.

### 3.9 Molecular docking studies of EG

The active site residues of COX-2 (PDB ID: 5IKT) were His90, Val116, Arg120, Phe205, Phe209, Val228, Tyr348, Val349, Leu352, Ser353, Tyr355, Leu359, Asn375, Ile377, Phe381, Leu384, Tyr385, Trp387, Phe518, Met522, Val523, Gly526, Ala527, Phe529, Ser530, Leu531, Gly533, and Leu534. In MMP-9 (PDB ID: 4HMA) the active site residues were Tyr179, Pro180, Gly186, Leu187, Leu188, Ala189, His190, Ala191, Phe192, Pro193, Leu222, Val223, His226, Gln227, His230, Gly233, Leu234, Asp235, His236, Ser237, Ser238, Pro240, Ala242, Leu243, Tyr245, Pro246, Met247, and Tyr248. Similarly, the active site residues in TNF-α (PDB ID: 2AZ5) were His15, Leu57, Ile58, Tyr59, Ser60, Tyr119, Leu120, Gly121, Gly122, Gln149, Val150, Tyr151, and Ile155.

**FIGURE 7:**
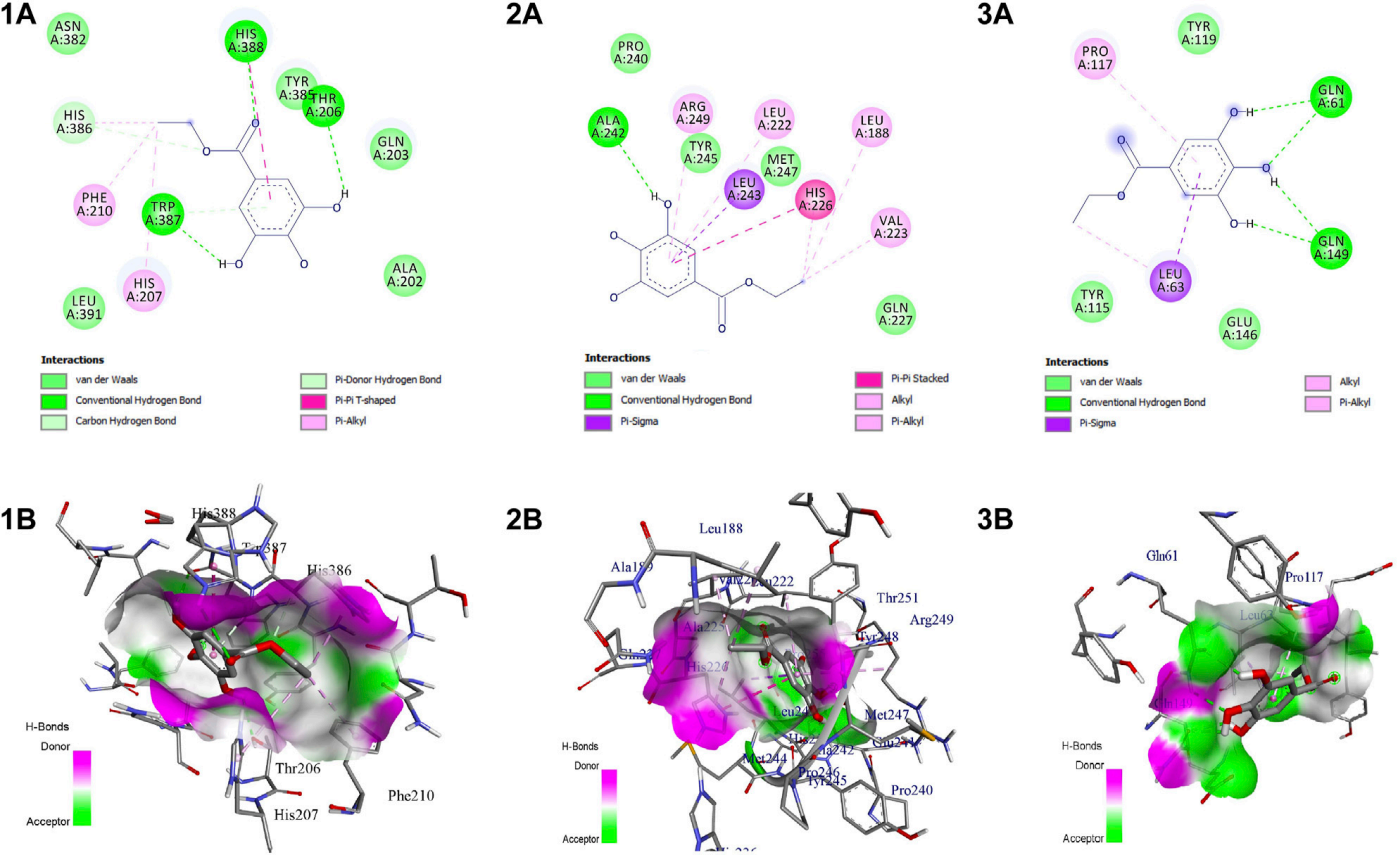
Intermolecular interaction of EG with (1) COX-2, (2) MMP-9, (3) TNF-α. **(A)** 2D representation, **(B)** representation of ligand within the binding pocket.

EG scored the lowest binding energy (BE) of -6.2 kcal/mol with COX-2 through the formation of five non-hydrogen bond interactions with Ala527 (2), Tyr355, Val523, and Leu352. The lowest BE of −4.6 kcal/mol was scored by EG with TNF-α by forming one hydrogen bond interaction with Ser60 and four non-hydrogen bond interactions with Leu57, Ile155, and Tyr59 (2). Likewise, the binding energy of EG with MMP-9 was predicted to be −7.2 kcal/mol through the formation of one hydrogen bond interaction with Ala242 and seven non-hydrogen bond interactions with Arg249, Leu222, Leu243, His226 (2), Val223, and Leu188. Figure 7 represents the intermolecular interaction of EG with COX-2, MMP-9, and TNF-α respectively.

#### 3.9.1 Molecular dynamics

##### 3.9.1.1 Stability of EG-COX-2 complex

**FIGURE 8:**
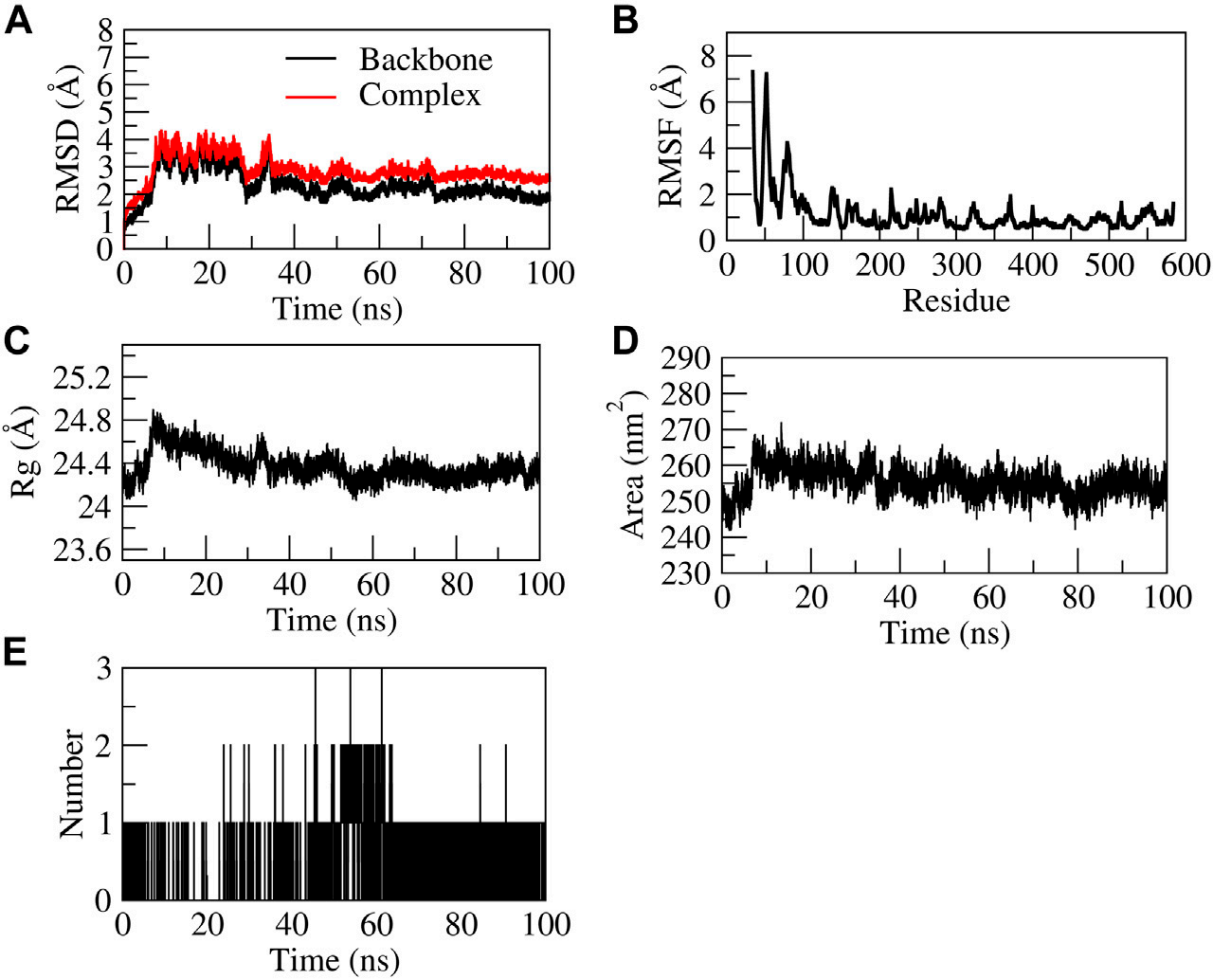
Structural stability of EG-COX-2 complex. **(A)** RMSD of backbone and complex, **(B)** Root mean square fluctuation (RMSF), **(C)** Rg, **(D)** Solvent accessible surface area (SASA), **(E)** number of H-bond interactions during 100 ns MD simulation.

The EG-COX-2 complex exhibited stable dynamics after an equilibration period of 35 ns. Initially, the backbone and the complex root mean square deviation (RMSD) values gradually increased from ∼1 to 4 Å respectively and further showed steady decrease in the RMSD up to 73 ns and showed stable RMSD throughout 100 ns MD simulation. The N-terminal residues showed larger fluctuations up to ∼8 Å and the residues Ala527, Tyr355, Val523, and Leu352 showed relatively fewer fluctuations (∼2.0 Å), as they participated in stable non-bonded interactions (Supplementary Movie S1). The compactness of the protein was studied by radius of gyration (Rg). Initially from 0 to 10 ns, a steady increase in Rg was observed up to 24.8 Å. Later, from 10 to 60 ns, it steadily decreased to ∼24.4 Å and was stable from 60 to 100 ns, which indicates the formation of a compact globular shape of the binding pocket and higher compactness of the complex. The initial and final surface area occupied by EG-COX-2 docked complex was ∼245 nm^2^ and 255 nm^2^. Interestingly, a similar trend in the surface area was observed to that of Rg and which indicates a decreased surface area of the binding pocket and the formation of a stable ligand-protein complex. The complex formed three H-bonds, of which one H-bond was consistent throughout the simulation. Figure 8 represents the stability of EG-COX-2 complex.

##### 3.9.1.2 Stability of EG-MMP-9 complex

**FIGURE 9:**
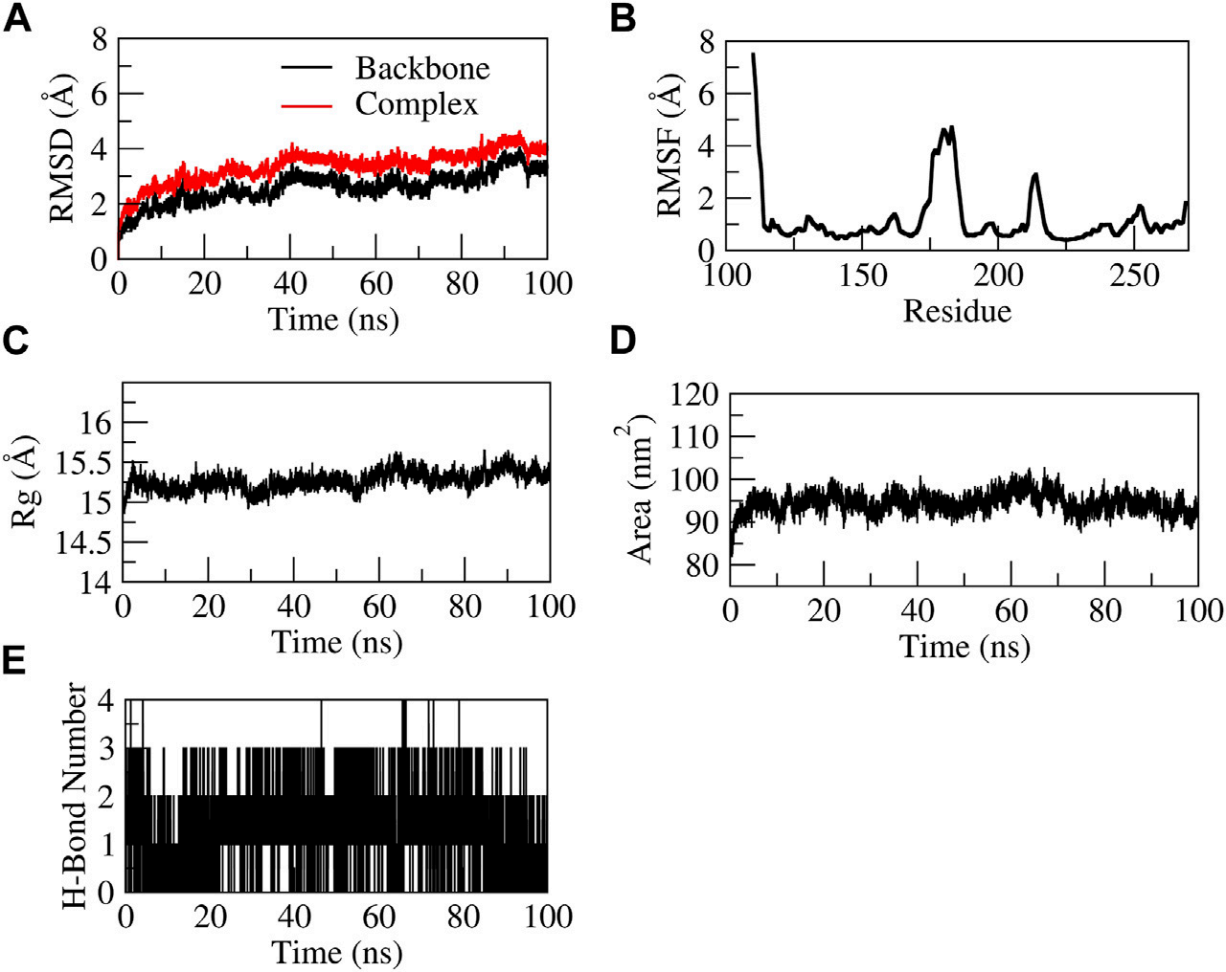
Structural stability of EG-MMP-9 complex. **(A)** RMSD of backbone and complex, **(B)** RMSF, **(C)** Rg, **(D)** SASA, **(E)** number of H-bond interactions during 100 ns MD simulation.

The EG-MMP-9 complex showed stability during 100 ns of stimulation after a 40 ns equilibration period. Initially the complex RMSD and backbone values gradually increased from 4.0 to 5.3 Å and from ∼1.0 to 3.5 Å, respectively. The N terminal residue showed the highest fluctuation up to 8Å and the residues Ala242, Arg249, Leu243, His226, and Val223 showed relatively fewer fluctuations (∼1.8 Å), as they participated in stable non-bonded interactions with MMP-9, while the residue Leu222 and Leu188 showed higher fluctuations (∼3.5 Å) (Supplementary Movie S2). In EG-MMP-9 complex, the protein was found to be stable at 15-15.5 Å from 20 to 100 ns. This indicates the formation of a compact globular shape of the binding pocket and the higher compactness of the complex. Similarly, the surface area was also found to be stable for MMP-9-EG complex occupying the surface area ∼90 and 100 nm^2^. The complex formed four H-bonds, of which two H-bonds were consistent throughout the simulation. Figure 9 represents the stability of EG-MMP-9 complex.

##### 3.9.1.3 Stability of EG-TNF-α complex

**FIGURE 10:**
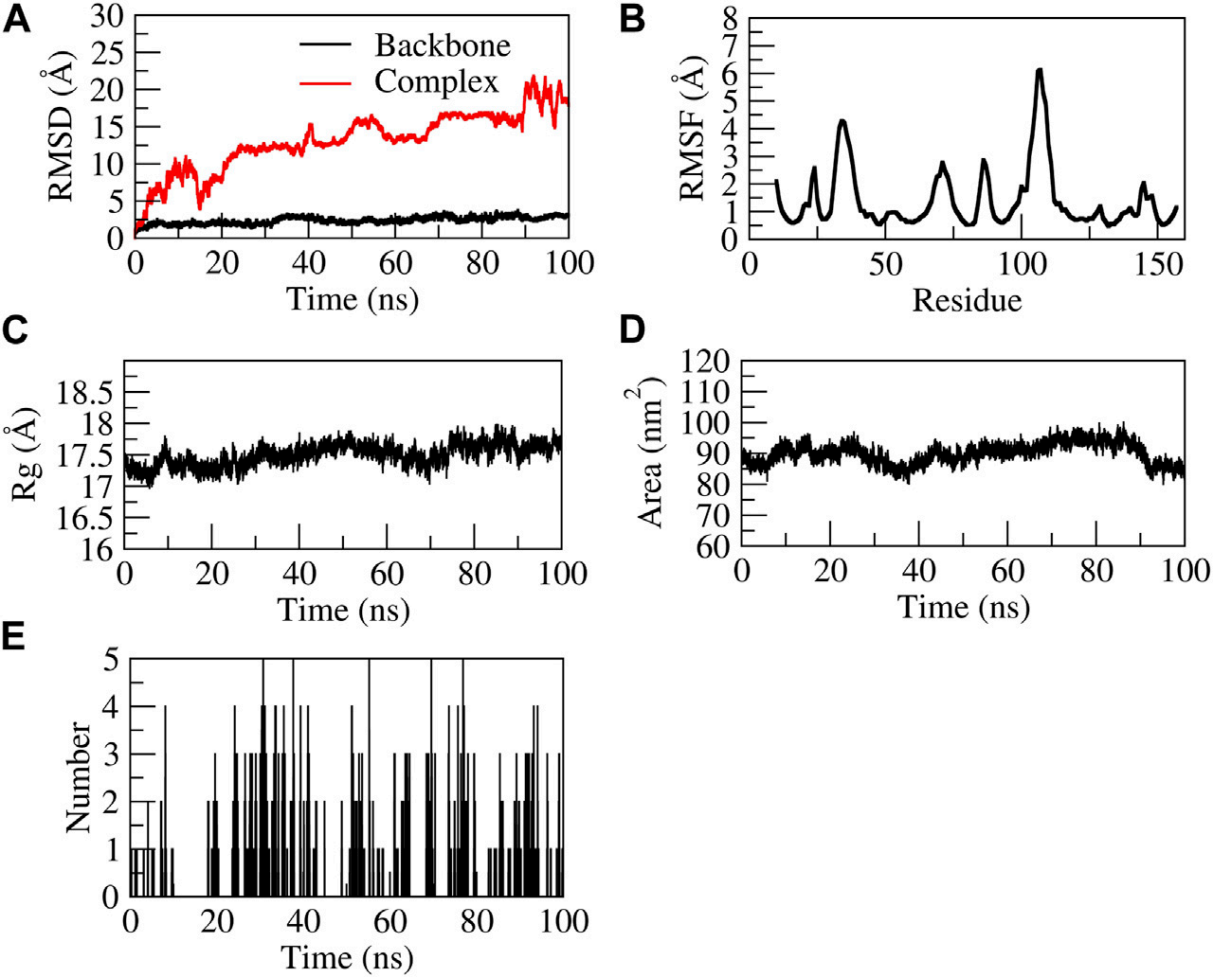
Structural stability of EG-TNF-α complex. **(A)** RMSD of backbone and complex, **(B)** RMSF, **(C)** Rg, **(D)** SASA, **(E)** number of H-bond interactions during 100 ns MD simulation.

The EG-TNF-α complex showed higher fluctuations during 100 ns of stimulation as compared to the backbone. The backbone showed stability throughout the stimulation process of 100 ns. The RMSD of the backbone was observed to be from 0 to 4. However, an increase in the RMSD value of the complex was observed from 0 to 25 Å. EG was found to be unstable with TNF-α as it moved out of the binding pocket (Supplementary Movie S3). The N terminal residue showed slight fluctuations up to 2Å and the residues Ser60, Leu57, Tyr59, and Ile155 showed relatively fewer fluctuations (∼1.8 Å) as they participated in stable non-bonded interactions with TNF-α. The residues aa30 to aa45 and aa105 to aa115 showed higher fluctuation. The compactness of the TNF-α protein was found to be stable at 17–17.5 Å from 20–100 ns. Similarly, the surface area was also found to be stable for TNF-α. The complex formed five H-bonds, of which none were consistent throughout the simulation due to unstable contacts of EG with TNF-α. Figure 10 represents the stability of EG-TNF-α complex.

## 4 Discussion

Wound healing is an important physiological process to restore the anatomical and functional integrity of injured skin tissue. This is regulated by various complex cellular and molecular pathways to induce proliferation, migration, and differentiation of cells and thereby affected tissue renovates at the earliest to its normal status (Bayrami et al., 2018). Even though the human body has an incredible capacity to heal wounds, the healing process depends on the nature and degree of damage, the reparative capability of the wounded tissues, and the health status of the human body (Kumara Swamy et al., 2007). However, metabolic syndromes and microbial infections interrupt the healing process and cause a considerable delay in the recovery of wounded tissues. Therefore, they necessitate the use of substances that give protection from unfavorable risk factors causing interruptions in wound healing and that have the capability to accelerate the re-epithelialization process without any side effects (Juszczak et al., 2022). In the last few decades, natural compounds obtained from traditional medicinal plants have been of great interest in drug discovery innovations. Their ability to interact with various molecular targets, and potent pharmacological properties with apparently fewer side effects, have made them indispensable in expediting the wound healing process (Elloumi et al., 2022).

*C. mimosoides* is one of the most important ethnomedicinal plants being utilized by the herbal healers of Thailand and India to treat multiple diseases, chiefly wounds and other skin-related ailments. We have previously evaluated the wound-healing activity of crude ethanol extract and its PEF (Bhat et al., 2016; Bhat et al., 2022), demonstrating both were successfully able to ameliorate the wound-healing process. In the current study, based on our previous findings, we carried out bioassay-guided fractionation of the most active sample PEF, with the aim of isolating and identifying the bioactive compound/s responsible for wound-healing activity. The known quantity of PEF was eluted in a chromatography column using combinations of eluents (CHCl_3_:MeOH) in different ratios, producing 24 fractions. Of them all, fraction nine exhibited a significantly higher percentage of cell migration in scratch wound assay with effective antimicrobial and antioxidant activities. Owing to its potential activities, fraction nine was re-fractionated to get sub-fractions which were again analyzed for scratch wound assay, followed by an antimicrobial activity. Among all the 15 sub-fractions, the sub-fraction 9.13 was the most active in promoting a higher percentage of cell migration followed by significant antimicrobial and antioxidant activities. The sub-fraction 9.13, containing only one dominant compound, was directly analyzed by various spectroscopic studies and further was confirmed as EG.

EG is an important natural polyphenolic compound reported in several plant species such as *Acacia nilotica* (L.) Delile (Kalaivani et al., 2011; Mohan et al., 2014), *Acalypha wilkesiana* var. *lace-acalypha* (Oladimeji and Igboasoiyi, 2014), *Caesalpinia pluviosa* DC. (Flores et al., 2006), *Dimocarpus longan* Lour (Tseng et al., 2014; Tang et al., 2019), *Euphorbia fischeriana* Steud (Cui et al., 2015), *Geranium carolinianum* L. (Ooshiro et al., 2009), *Lagerstroemia speciosa* (L.) Pers. (Gao et al., 2010), *Libidibia ferrea* (Mart. ex Tul.) L.P.Queiroz (Passos et al., 2021), *Phyllanthus urinaria* L. (Paulino et al., 1999), *Pistacia integerrima* J. L. Stewart ex Brandis (Mehla et al., 2011), *Terminalia arjuna* (Roxb. ex DC.) Wight & Arn. (Pettit et al., 1996), and *Terminalia chebula* Retz (Saleem et al., 2002; Li et al., 2016). We reported EG in crude ethanol extract and its PEF of *C. mimosoides* for the first time with 56.88 and 255.91 μg/ml concentrations respectively (Bhat et al., 2016; Bhat et al., 2022). Previous studies have shown that EG is widely used as a food additive due to its high antioxidative nature (Hall et al., 1996; Kalaivani et al., 2011; Kim et al., 2012; Mohan et al., 2014; Mohan et al., 2014; Mohan et al., 2015; Mohan et al., 2017) and it shows protective effects against septic shock (Mink et al., 2011). It was also reported to have anticancer (Pettit et al., 1996; Kim et al., 2012; Mohan et al., 2014; Mohan et al., 2015; Mohan et al., 2017), antimicrobial (Ooshiro et al., 2009; Soe et al., 2011; Oladimeji and Igboasoiyi, 2014; Tseng et al., 2014; Li et al., 2016; Tang et al., 2019), muscle relaxant (Paulino et al., 1999), and anti-inflammatory (Murase et al., 1999; Mehla et al., 2011; Park et al., 2011; Mehla et al., 2013) activities.

Here we reported the isolation and characterization of EG in *C. mimosoides* through bioassay-guided fractionation and isolation procedure, followed by its wound-healing activity for the first time. More than 100% viability of cells was recorded at ≤7.81 μg/ml dose and the cell toxicity (IC_50_) was determined at 104.08 ± 1.19 μg/ml concentration. This is predominantly within the recommended threshold (toxicity level: IC_50_ ≤ 30 μg/ml) set by the American National *Cancer* Institute (ANCI) (Bolla et al., 2019), which provides the safety margin of EG to carry out further *in vitro* scratch wound assay. The study demonstrated that EG significantly stimulated the rate of migration of L929 cells to the wound site with 92.09 ± 0.19 and 97.98 ± 0.46%, at lower doses (1.95 and 3.90 μg/ml respectively). The stimulation of cell migration at 3.90 μg/ml dose of EG was nearly similar to that of the positive control (EGF). The study revealed that *in vitro* wound-healing efficacy of EG was comparatively superior over PEF (the fraction containing EG), in which the cell migration rate in PEF was 89.16 ± 1.98% and 92.59 ± 1.53% in L929 cells, at 3.90 and 7.81 μg/ml doses respectively at the 48th hour of incubation period (Bhat et al., 2022). Previous studies have reported that the migration of fibroblast cells to the wound site is linked with the antioxidant property of the treated molecules (Juszczak et al., 2022). In the present study, this hypothesis holds good for sub-fraction 13 and its major component EG, which have exhibited highly significant antioxidant activities (DPPH and ABTS), almost equal to their corresponding standards. A similar mode of cell migration was also reported in *Lavandula stoechas* L. extract containing luteolin derivatives, which exhibited prominent stimulation of fibroblast cells at lower concentrations (54.49 ± 5.02 mg/L), and the activity was decreased with increased concentrations of the sample (Addis et al., 2020). Similarly, the compounds longiferone B, pinostrobin, dihydro bisdemethoxycurcumin, and β-sitosterol-D-glucoside isolated from *Boesenbergia kingii* Mood and L.M. Prince enhanced the migration of L929 cells to 60%–76% at 10 μM concentrations on the second day of incubation (Sudsai et al., 2016). On the other hand, Sarkhail et al. (2020) reported a 45% reduction of scratch wound in NIH/3T3 murine fibroblast cells after 48 h of incubation when they were treated with 3-epimasticadienolic acid isolated from *Pistacia vera* L. at concentrations higher than 200 μg/ml.

Polyphenols are the major constituents of plant metabolites, considered as an essential element in the human healthcare system. Currently, the scientific interest towards the treatment of wound complications using natural polyphenolic compounds is increasing rapidly (Yadav et al., 2018). In earlier studies, various phenolic compounds with potential wound-healing activity were isolated through a bioassay-guided fractionation procedure. Compounds such as apigenin, daphnetin, demethyldaphnoretin 7-O-glucoside, (-)-epicatechin, hypericin, hyperoside, isoquercitrin, kaempferol, luteolin, myricetin-3-*O*-rhamnoside, quercetin-3-*O*-rhamnoside, and rutin (Süntar et al., 2010; Süntar et al., 2010; Süntar et al., 2012; Süntar et al., 2013; Karakaya et al., 2020; Elloumi et al., 2022) were isolated from different plant origins and have been repeatedly proven to accelerate the process of wound healing both in *vitro* and *in vivo* models. The curative properties of these phenolic compounds are mainly attributed to their effective antimicrobial, anti-inflammatory, and antioxidant activities, which are positively correlated with each other. They play a significant role in enhancing the healing activity by forming a barrier against contagious microbes to alleviate possible wound infections and to protect the cells from being damaged by reactive oxygen species (ROS) (Yadav et al., 2018; Baidoo et al., 2021). Nevertheless, concurrent to these studies, the current invention exhibited a significantly enhanced rate of wound contraction in both circular excision and longitudinal incision wounds treated with ointments containing 1% EG. It might have possibly correlated with its effective antimicrobial activity with the least MIC values against wound-invading microbes such as *P. aeruginosa*, *S. aureus*, *M. luteus*, *P. vulgaris*, and *M. flavus*. At the same time, EG exhibited efficient antioxidant activity with significantly lower IC_50_ values against DPPH and ABTS radicals. The potential wound-healing activity of 1% EG ointment was also clearly evidenced by tensile strength, histopathology, and biochemical parameters of granulation tissues on the 15th post-wounding day. According to Mekonnen et al. (2013), the contraction and complete epithelialization of wounds with increased tensile strength in a shorter period is a characteristic feature of effective wound management. In support of this, EG at 1% ointment successfully facilitated the proliferation and migration of fibroblasts, keratinocytes, and epithelial cells, followed by the formation of matured collagen fibers at the wound site to restore skin integrity with higher tensile strength, compared to other treatment groups. The epithelial layer was found well oriented with 2–3 cells in thickness, covered by a thick keratin layer. The dermal region was well orchestrated with an accomplished process of angiogenesis, maximum cellular infiltration, transformation of fibroblasts into myofibroblasts, prominent blood vessels, increased mast cells, distribution of scattered inflammatory cells, and reduction in macrophage and neutrophil cell counts as judged by H&E, Massons trichome, and Toluidine blue-stained sections.

Hydroxyproline and hexosamine are vital components of collagen fiber and hence the measurement of these two contents in granular tissue is a key indicator for collagen turnover (Dwivedi et al., 2016). In the present study, higher contents of hydroxyproline (42.92 ± 2.12 mg/g) and hexosamine (8.47 ± 0.60 mg/g) in the 1% EG ointment-treated group might have played a significant role in the speedy recovery of wounds with shorter epithelialization period. Similarly, an increased quantity of cellular antioxidant elements (SOD, CAT, and GSH) and down-regulation of oxidative stress-mediated marker (LPO) at the significant rate might have played a crucial role in the elevation of wound contraction in 1% EG ointment-treated groups.

It is a notable fact that normal levels of pro-inflammatory cytokines prevent infection and accelerate the normal wound-healing process. However, excessive production of inflammatory markers is detrimental to successful wound healing as it leads to prolonged inflammatory conditions (Mehla et al., 2011). Thus, counteracting the overproduction of pro-inflammatory cytokines endorses a therapeutic effect in chronic wound healing. In earlier studies, EG was successfully reported to alleviate the inflammatory condition in acute lung injury through a significant reduction in pro-inflammatory cytokines by blocking AP-1 transcription factor, followed by inhibiting Nf-kB activation with increased HO-1/Nrf_2_ signaling pathways (Murase et al., 1999; Mehla et al., 2011; Mehla et al., 2013). In our study, the effective wound-healing activity of EG was also supported by *in silico* studies. The docking parameter was performed for EG against the protein targets of inflammatory markers such as COX-2, MMP-9, and TNF-α. The mode of binding predicted by the docking studies established a connection with the affinity of binding and the biological activity of the constituent. Previous studies carried out by Wang et al. (2018) hypothesized that the effective pro-inflammatory activity of *D. longan* fruits in cell lines and experimental animals might be attributed to the activation of COX-1 and COX-2 peroxidase active sites stimulated by EG. The molecular dynamic studies elucidated its competitive binding mode and stability of the complex with COX-2. EG at C-3-OH or C-4-OH site markedly shortened the distance between Fe^4+^ and the respective O^−^and thereby increased the binding affinity. Further, Cui et al. (2015) demonstrated the modulating capacity of EG through PI3K/Akt signaling pathways by inhibiting MMP-9 and MMP-2 mRNA levels and the downstream targets such as NF-*κ*B p-65 and Bcl-2/Bax. Our finding is in line with previous reports and EG was found to bind stably with the active site residues of MMP-9. However, on looking into TNF-α, EG was unstable throughout 100 ns MD simulation, which supports EG as a potent inhibitor for inflammatory markers and playing a crucial role in the wound-healing process.

## 5 Conclusion

Using a bioassay-guided fractionation procedure, we successfully isolated an active natural antioxidant compound, EG, from PEF of *C. mimosoides*. The compound displayed a remarkable wound-healing activity with its enhanced antimicrobial and anti-inflammatory properties. The study highlighted the role of EG, which could facilitate the proliferation of extracellular matrix components such as fibroblasts, keratinocytes, epithelial cells, blood vessels, and collagen fibers, which are known to contribute to successful re-epithelialization of wounded tissues. Additionally, the significant up-regulation of enzymatic and non-enzymatic cellular antioxidant elements and significant down-regulation of oxidative stress-mediated markers endorsed the potentiality of EG in the wound-healing process. Molecular docking analysis of EG against different molecular targets predicted the possible mechanism of action in wound-healing activity. Our study successfully validated the wound-healing efficacy of EG and supports the traditional applications of *C. mimosoides* plant in wound treatments. Further, these findings pave the way for molecular mechanistic studies and clinical investigations that could enable the use of EG as a promising natural remedy for the treatment of cutaneous wounds.

## Data Availability

The datasets presented in this study can be found in online repositories. The names of the repository/repositories and accession number(s) can be found in the article/Supplementary Material.

## References

[B1] Addis R. Cruciani S. Santaniello S. Bellu E. Sarais G. Ventura C. (2020). Fibroblast proliferation and migration in wound healing by phytochemicals: Evidence for a novel synergic outcome. Int. J. Med. Sci. 17, 1030–1042. 10.7150/ijms.43986 32410832 PMC7211158

[B2] Amri B. Martino E. Vitulo F. Corana F. Kaâb L. B. Rui M. (2017). Marrubium vulgare L. Leave extract: Phytochemical composition, antioxidant and wound healing properties. Molecules 22, 1851. 10.3390/molecules22111851 29143793 PMC6150401

[B3] Antoni C. Vera L. Devel L. Catalani M. P. Czarny B. Cassar-Lajeunesse E. (2013). Crystallization of bi-functional ligand protein complexes. J. Struct. Biol. 182, 246–254. 10.1016/j.jsb.2013.03.015 23567804

[B4] Azis H. A. Taher M. Ahmeda A. S. Sulaiman W. M. A. W. Susanti D. Chowdhury S. R. (2017). *In vitro* and *in vivo* wound healing studies of methanolic fraction of *Centella asiatica* extract. S Afr. J. Bot. 108, 163–174. 10.1016/j.sajb.2016.10.022

[B5] Baidoo M. F. Mensah A. Y. Ossei P. P. S. Asante-Kwatia E. Amponsah I. K. (2021). Wound healing, antimicrobial and antioxidant properties of the leaf and stem bark of *Entada africana* . Guill. Perr. *S Afr J Bot* 137, 52–59. 10.1016/j.sajb.2020.09.037

[B6] Bayrami Z. Hajiaghaee R. Khalighi-Sigaroodi F. Rahimi R. Farzaei M. H. Hodjat M. (2018). Bio-guided fractionation and isolation of active component from *Tragopogon graminifolius* based on its wound healing property. J. Ethnopharmacol. 226, 48–55. 10.1016/j.jep.2018.08.002 30096362

[B7] Bhat P. B. Hegde S. Upadhya V. Hegde G. R. Habbu P. V. Mulgund G. S. (2016). Evaluation of wound healing property of *Caesalpinia mimosoides* Lam. J. Ethnopharmacol. 193, 712–724. 10.1016/j.jep.2016.10.009 27717906

[B8] Bhat P. Hegde G. Hegde G. R. (2012). Ethnomedicinal practices in different communities of Uttara Kannada district of Karnataka for treatment of wounds. J. Ethnopharmacol. 143, 501–514. 10.1016/j.jep.2012.07.003 22820243

[B9] Bhat P. Hegde G. R. Hegde G. Mulgund G. S. (2014). Ethnomedicinal plants to cure skin diseases-An account of the traditional knowledge in the coastal parts of Central Western Ghats, Karnataka, India. J. Ethnopharmacol. 151, 493–502. 10.1016/j.jep.2013.10.062 24239890

[B10] Bhat P. Upadhya V. Hegde G. R. Hegde H. V. Roy S. (2022). Attenuation of dermal wounds through topical application of ointment containing phenol enriched fraction of *Caesalpinia mimosoides* Lam. Front. Pharmacol. 13, 1025848. 10.3389/fphar.2022.1025848 36313327 PMC9608657

[B11] Biswas D. Yoganandam G. P. Dey A. Deb L. (2013). Evaluation of antimicrobial and wound healing potentials of ethanol extract of *Wedelia biflora* Linn D.C. leaves. Indian J. Pharm. Sci. 75, 156–161. 24019563 PMC3757853

[B12] Bolla S. R. Mohammed Al-Subaie A. Yousuf Al-Jindan R. Papayya Balakrishna J. Kanchi Ravi P. Veeraraghavan V. P. (2019). *In vitro* wound healing potency of methanolic leaf extract of *Aristolochia saccata* is possibly mediated by its stimulatory effect on collagen-1 expression. Heliyon 20, e01648. 10.1016/j.heliyon.2019.e01648 PMC6529694 31193473

[B13] Chanwitheesuk A. Teerawutgulrag A. Kilburn J. D. Rakariyatham N. (2007). Antimicrobial gallic acid from *Caesalpinia mimosoides* Lamk. Food Chem. 100, 1044–1048. 10.1016/j.foodchem.2005.11.008

[B14] Charan J. Kantharia N. D. (2013). How to calculate sample size in animal studies? J. Pharmacol. Pharmacother. 4, 303–306. 10.4103/0976-500X.119726 24250214 PMC3826013

[B15] Cui H. Yuan J. Du X. Wang M. Yue L. Liu J. (2015). Ethyl gallate suppresses proliferation and invasion in human breast cancer cells via Akt-NF-κB signaling. Oncol. Rep. 33, 1284–1290. 10.3892/or.2014.3682 25522911

[B16] DasNandy A. Patil V. S. Hegde H. V. Harish D. R. Roy S. (2022). Elucidating type 2 diabetes mellitus risk factor by promoting lipid metabolism with gymnemagenin: An *in vitro* and *in silico* approach. Front. Pharmacol. 13, 1074342. 10.3389/fphar.2022.1074342 36582536 PMC9792475

[B17] Dwivedi D. Dwivedi M. Malviya S. Singh V. (2016). Evaluation of wound healing, anti-microbial and antioxidant potential of *Pongamia pinnata* in wistar rats. J. Tradit. Complement. Med. 7, 79–85. 10.1016/j.jtcme.2015.12.002 28053891 PMC5198820

[B18] Elloumi W. Mahmoudi A. Ortiz S. Boutefnouchet S. Chamkha M. Sayadi S. (2022). Wound healing potential of quercetin-3-O-rhamnoside and myricetin-3-O-rhamnoside isolated from *Pistacia lentiscus* distilled leaves in rats model. Biomed. Pharmacother. 146, 112574. 10.1016/j.biopha.2021.112574 35062055

[B19] Flores Y. Vila J. Almanza G. R. (2006). Secondary metabolites from *Caesalpinia fluviosa* . Rev. Boliv. Quím. 23, 1–8.

[B20] Gao S. Zhan Q. Li J. Yang Q. Li X. Chen W. (2010). LC-MS/MS method for the simultaneous determination of ethyl gallate and its major metabolite in rat plasma. Biomed. Chromatogr. 24, 472–478. 10.1002/bmc.1314 19688816

[B21] Hall G. Le T. T. T. Stanford J. B. Sugden J. K. (1996). Hydroxyl radical scavenging by ethyl gallate and related compounds: A method for rapid evaluation. Pharm. acta Helv. 71, 221–224. 10.1016/0031-6865(96)00013-1

[B22] Hashemnia M. Nikousefat Z. Mohammadalipour A. Zangeneh M. Zangeneh A. (2019). Wound healing activity of *Pimpinella anisum* methanolic extract in streptozotocin-induced diabetic rats. J. Wound Care 28, S26–S36. 10.12968/jowc.2019.28.sup10.s26 31600102

[B23] He M. M. Smith A. S. Oslob J. D. Flanagan W. M. Braisted A. C. Whitty A. (2005). Small-molecule inhibition of TNF-alpha. Science 310, 1022–1025. 10.1126/science.1116304 16284179

[B24] Juszczak A. M. Jakimiuk K. Czarnomysy R. Strawa J. W. Zovko Končić M. Bielawski K. (2022). Wound healing properties of *Jasione montana* extracts and their main secondary metabolites. Front. Pharmacol. 13, 894233. 10.3389/fphar.2022.894233 35620288 PMC9127232

[B25] Kalaivani T. Rajasekaran C. Mathew L. (2011). Free radical scavenging, cytotoxic and hemolytic activities of an active antioxidant compound ethyl gallate from leaves of *Acacia nilotica* (L) Wild ex Delile subsp *indica* (Benth) Brenan. J. Food Sci. 76, T144–T149. 10.1111/j.1750-3841.2011.02243.x 22417526

[B26] Karakaya S. Süntar I. Yakinci O. F. Sytar O. Ceribasi S. Dursunoglu B. (2020). *In vivo* bioactivity assessment on *epilobium* species: A particular focus on *Epilobium angustifolium* and its components on enzymes connected with the healing process. J. Ethnopharmacol. 262, 113207. 10.1016/j.jep.2020.113207 32730870

[B27] Kasouni A. I. Chatzimitakos T. G. Stalikas C. D. Trangas T. Papoudou-Bai A. Troganis A. N. (2021). The unexplored wound healing activity of*Urtica dioica* L. extract: An *in vitro* and *in vivo* study. Molecules 26, 6248. 10.3390/molecules26206248 34684829 PMC8540079

[B28] Khanal P. Patil V. Bhandare V. V. Patil P. P. Patil B. M. Dwivedi P. S. (2023). Systems and *in vitro* pharmacology profiling of diosgenin against breast cancer. Front. Pharmacol. 13, 1052849. 10.3389/fphar.2022.1052849 36686654 PMC9846155

[B29] Khanal P. Patil V. S. Bhandare V. V. Dwivedi P. S. Shastry C. S. Patil B. M. (2022). Computational investigation of benzalacetophenone derivatives against SARS-CoV-2 as potential multi-target bioactive compounds. Comput. Biol. Med. 146, 105668. 10.1016/j.compbiomed.2022.105668 35667894 PMC9135652

[B30] Kim W. H. Song H. O. Choi H. J. Bang H. I. Choi D. Y. Park H. (2012). Ethyl gallate induces apoptosis of HL-60 cells by promoting the expression of caspases-8, -9, -3, apoptosis-inducing factor and endonuclease G. Int. J. Mol. Sci. 13, 11912–11922. 10.3390/ijms130911912 23109891 PMC3472783

[B31] Krivák R. Hoksza D. (2018). P2Rank: Machine learning based tool for rapid and accurate prediction of ligand binding sites from protein structure. J. cheminform 10, 39. 10.1186/s13321-018-0285-8 30109435 PMC6091426

[B32] Kumara Swamy H. M. Krishna V. Shankarmurthy K. Abdul Rahiman B. Mankani K. L. Mahadevan K. M. (2007). Wound healing activity of embelin isolated from the ethanol extract of leaves of *Embelia ribes* Burm. J. Ethnopharmacol. 109, 529–534. 10.1016/j.jep.2006.09.003 17034970

[B33] Kumari A. Raina N. Wahi A. Goh K. W. Sharma P. Nagpal R. (2022). Wound-healing effects of curcumin and its nano formulations: A comprehensive review. Pharmaceutics 14, 2288. 10.3390/pharmaceutics14112288 36365107 PMC9698633

[B34] Li K. Lin Y. Li B. Pan T. Wang F. Yuan R. (2016). Antibacterial constituents of *Fructus chebulae immaturus* and their mechanisms of action. BMC Complement. Altern. Med. 16, 183. 10.1186/s12906-016-1162-5 27368700 PMC4930599

[B35] Manasa M. Vivek M. N. Kambar Y. Ramesh Kumar K. A. Prashith Kekuda T. R. (2014). Mineral content, antimicrobial and radical scavenging potential of *Caesalpinia mimosoides* Lamk (Caesalpiniaceae). World J. Pharm. Res. 3, 1047–1063.

[B36] Mehla K. Balwani S. Agrawal A. Ghosh B. (2013). Ethyl gallate attenuates acute lung injury through Nrf2 signaling. Biochimie 95, 2404–2414. 10.1016/j.biochi.2013.08.030 24018486

[B37] Mehla K. Balwani S. Kulshreshtha A. Nandi D. Jaisankar P. Ghosh B. (2011). Ethyl gallate isolated from *Pistacia integerrima* Linn. inhibits cell adhesion molecules by blocking AP-1 transcription factor. J. Ethnopharmacol. 137, 1345–1352. 10.1016/j.jep.2011.07.068 21843619

[B38] Mekonnen A. Sidamo T. Asres K. Engidawork E. (2013). *In vivo* wound healing activity and phytochemical screening of the crude extract and various fractions of *Kalanchoe petitiana* A. Rich (Crassulaceae) leaves in mice. J. Ethnopharmacol. 145, 638–646. 10.1016/j.jep.2012.12.002 23228912

[B39] Mink S. N. Jacobs H. Gotes J. Kasian K. Cheng Z. Q. (2011). Ethyl gallate, a scavenger of hydrogen peroxide that inhibits lysozyme-induced hydrogen peroxide signaling *in vitro*, reverses hypotension in canine septic shock. J. Appl. Physiol. 110, 359–374. 10.1152/japplphysiol.00411.2010 21071593

[B40] Mohan S. Thiagarajan K. Chandrasekaran R. Arul J. (2014). *In vitro* protection of biological macromolecules against oxidative stress and *in vivo* toxicity evaluation of *Acacia nilotica* (L) and ethyl gallate in rats. BMC Complement. Altern. Med. 14, 257. 10.1186/1472-6882-14-257 25043389 PMC4223376

[B41] Mohan S. Thiagarajan K. Chandrasekaran R. (2017). Evaluation of ethyl gallate for its antioxidant and anticancer properties against chemical-induced tongue carcinogenesis in mice. Biochem. J. 474, 3011–3025. 10.1042/BCJ20170316 28679629

[B42] Mohan S. Thiagarajan K. Chandrasekaran R. (2015). *In vitro* evaluation of antiproliferative effect of ethyl gallate against human oral squamous carcinoma cell line KB. Nat. Prod. Res. 29, 366–369. 10.1080/14786419.2014.942303 25104086

[B43] Moyo P. Kunyane P. Selepe M. A. Eloff J. N. Niemand J. Louw A. I. (2019). Bioassay-guided isolation and identification of gametocytocidal compounds from *Artemisia afra* (Asteraceae). Malar. J. 18, 65. 10.1186/s12936-019-2694-1 30849984 PMC6408838

[B44] Muhammad S. L. Wada Y. Mohammed M. Ibrahim S. Musa K. Y. Olonitola O. S. (2021). Bioassay-guided identification of bioactive compounds from*Senna alata* L. against methicillin-resistant *Staphylococcus aureus* . Appl. Microbiol. 1, 520–536. 10.3390/applmicrobiol1030034

[B45] Murase T. Kume N. Hase T. Shibuya Y. Nishizawa Y. Tokimitsu I. (1999). Gallates inhibit cytokine-induced nuclear translocation of NF-kappaB and expression of leukocyte adhesion molecules in vascular endothelial cells. Arterioscler. Thromb. Vasc. Biol. 19, 1412–1420. 10.1161/01.atv.19.6.1412 10364071

[B46] Najmi A. Javed S. A. Al Bratty M. Alhazmi H. A. (2022). Modern approaches in the discovery and development of plant-based natural products and their analogues as potential therapeutic agents. Molecules 27, 349. 10.3390/molecules27020349 35056662 PMC8779633

[B47] Negut I. Grumezescu V. Grumezescu A. M. (2018). Treatment strategies for infected wounds. Molecules 23, 2392. 10.3390/molecules23092392 30231567 PMC6225154

[B48] OECD (2017). Test No 402: Acute dermal toxicity, OECD guidelines for the testing of chemicals, section 4. Paris: OECD Publishing. 10.1787/9789264070585-en

[B49] Oladimeji A. Igboasoiyi C. (2014). Isolation, characterization and antimicrobial analysis of ethyl gallate and pyrogallol from *Acalypha wilkesiana* var *lace-acalypha* (Muell & Arg). Afr. J. Pharmacol. Ther. 3, 79–84.

[B50] Oluwole D. O. Coleman L. Buchanan W. Chen T. La Ragione R. M. Liu L. X. (2022). Antibiotics-free compounds for chronic wound healing. Pharmaceutics 14, 1021. 10.3390/pharmaceutics14051021 35631606 PMC9143489

[B51] Ooshiro A. Hiradate S. Kawano S. Takushi T. Fujii Y. Natsume M. (2009). Identification and activity of ethyl gallate as an antimicrobial compound produced by *Geranium carolinianum* . Weed Biol. Manag. 9, 169–172. 10.1111/j.1445-6664.2009.00335.x

[B52] Orlando B. J. Malkowski M. G. (2016). Substrate-selective inhibition of cyclooxygeanse-2 by fenamic acid derivatives is dependent on peroxide tone. J. Biol. Chem. 291, 15069–15081. 10.1074/jbc.m116.725713 27226593 PMC4946924

[B53] Özbilgin S. Acıkara Ö. B. Akkol E. K. Süntar I. Keleş H. İşcan G. S. (2018). *In vivo* wound-healing activity of *Euphorbia characias* subsp. *wulfenii*: Isolation and quantification of quercetin glycosides as bioactive compounds. J. Ethnopharmacol. 224, 400–408. 10.1016/j.jep.2018.06.015 29920357

[B54] Pandey A. Kaushik A. Wanjari M. Dey Y. N. Jaiswal B. S. Dhodi A. (2017). Antioxidant and anti-inflammatory activities of *Aerva pseudotomentosa* leaves. Pharm. Biol. 55, 1688–1697. 10.1080/13880209.2017.1321022 28454506 PMC7011845

[B55] Park P. H. Hur J. Kim Y. C. An R. B. Sohn D. H. (2011). Involvement of heme oxygenase-1 induction in inhibitory effect of ethyl gallate isolated from *Galla Rhois* on nitric oxide production in RAW 264.7 macrophages. Arch. Pharm. Res. 34, 1545–1552. 10.1007/s12272-011-0917-2 21975817

[B56] Passos M. R. Almeida R. S. Lima B. O. Rodrigues J. Z. S. Macêdo Neres N. S. Pita L. S. (2021). Anticariogenic activities of *Libidibia ferrea*, gallic acid and ethyl gallate against *Streptococcus mutans* in biofilm model. J. Ethnopharmacol. 274, 114059. 10.1016/j.jep.2021.114059 33794333

[B57] Patil V. S. Harish D. R. Vetrivel U. Deshpande S. H. Khanal P. Hegde H. V. (2022). Pharmacoinformatics analysis reveals flavonoids and diterpenoids from *Andrographis paniculata* and *Thespesia populnea* to target hepatocellular carcinoma induced by Hepatitis B virus. Appl. Sci. 12, 10691. 10.3390/app122110691

[B58] Paulino N. Pizollatti M. G. Yunes R. A. Filho V. C. Creczynski-Pasa T. B. Calixto J. B. (1999). The mechanisms underlying the relaxant effect of methyl and ethyl gallates in the Guinea pig trachea *in vitro*: Contribution of potassium channels. Naunyn Schmiedeb. Arch. Pharmacol. 360, 331–336. 10.1007/s002109900081 10543436

[B59] Pettit G. R. Hoard M. S. Doubek D. L. Schmidt J. M. Pettit R. K. Tackett L. P. (1996). Antineoplastic agents 338. The cancer cell growth inhibitory constituents of *Terminalia arjuna* (Combretaceae). J. Ethnopharmacol. 53, 57–63. 10.1016/S0378-8741(96)01421-3 8844460

[B60] Rekha A. S. (2011). Pharmacognostical & experimental study on *Caesalpinia mimosoides* Lam – a folk plant. Bangalore, Karnataka, India: Dissertation submitted to the Rajiv Gandhi University of Health Sciences.

[B61] Saleem A. Husheem M. Härkönen P. Pihlaja K. (2002). Inhibition of cancer cell growth by crude extract and the phenolics of *Terminalia chebula* Retz. fruit. J. Ethnopharmacol. 81, 327–336. 10.1016/s0378-8741(02)00099-5 12127233

[B62] Santos T. S. Santos I. D. D. D. Pereira-Filho R. N. Gomes S. V. F. Lima-Verde I. B. Marques M. N. (2021). Histological evidence of wound healing improvement in rats treated with oral administration of hydroalcoholic extract of*Vitis labrusca* . Curr. Issues Mol. Biol. 43, 335–352. 10.3390/cimb43010028 34208147 PMC8929082

[B63] Sarkhail P. Navidpour L. Rahimifard M. Hosseini N. M. Souri E. (2020). Bioassay-guided fractionation and identification of wound healing active compound from *Pistacia vera* L. hull extract. J. Ethnopharmacol. 248, 112335. 10.1016/j.jep.2019.112335 31654800

[B64] Silja V. P. Varma S. K. Mohanan K. V. (2008). Ethnomedicinal plant knowledge of Mullu kuruma tribe of Waynad district, Kerala. Indian J. Tradit. Know 7, 604–612.

[B65] Soe W. M. Myint N. L. Sing L. C. Sakharkar M. K. Tang T. H. Sakharkar K. R. (2011). Ethyl gallate as a combination drug can overcome resistance in MRSA. Lett. Drug Des. Discov. 8, 65–68. 10.2174/157018011793663949

[B66] Sudsai T. Wattanapiromsakul C. Tewtrakul S. (2016). Wound healing property of isolated compounds from *Boesenbergia kingii* rhizomes. J. Ethnopharmacol. 26, 42–48. 10.1016/j.jep.2016.03.001 26945979

[B67] Süntar I. Küpeli Akkol E. Keles H. Yesilada E. Sarker S. D. Arroo R. (2012). Efficacy of *Daphne oleoides* subsp. *kurdica* used for wound healing: Identification of active compounds through bioassay guided isolation technique. J. Ethnopharmacol. 141, 1058–1070. 10.1016/j.jep.2012.04.001 22521733

[B68] Süntar I. Küpeli Akkol E. Keles H. Yesilada E. Sarker S. D. (2013). Exploration of the wound healing potential of *Helichrysum graveolens* (bieb) sweet: Isolation of apigenin as an active component. J. Ethnopharmacol. 149, 103–110. 10.1016/j.jep.2013.06.006 23764736

[B69] Süntar I. P. Akkol E. K. Yilmazer D. Baykal T. Kirmizibekmez H. Alper M. (2010). Investigations on the *in vivo* wound healing potential of *Hypericum perforatum* L. J. Ethnopharmacol. 127, 468–477. 10.1016/j.jep.2009.10.011 19833187

[B70] Tang Y. Y. He X. M. Sun J. Li C. B. Li L. Sheng J. F. (2019). Polyphenols and alkaloids in byproducts of longan fruits (*Dimocarpus Longan* Lour) and their bioactivities. Molecules 24, 1186. 10.3390/molecules24061186 30917573 PMC6471414

[B71] Tangsaengvit N. Kitphati W. Tadtong S. Bunyapraphatsara N. Nukoolkarn V. (2013). Neurite outgrowth and neuroprotective effects of quercetin from *Caesalpinia mimosoides* Lamk. on cultured P19-derived neurons. Evid. Based Complement. Altern. Med. 2013, 838051–838057. 10.1155/2013/838051 PMC3693115 23840266

[B72] Tian W. Chen C. Lei X. Zhao J. Liang J. (2018). CASTp 3.0: Computed atlas of surface topography of proteins. Nucleic Acids Res. 46, W363–W367. 10.1093/nar/gky473 29860391 PMC6031066

[B73] Tseng H. C. Wu W. T. Huang H. S. Wu M. C. (2014). Antimicrobial activities of various fractions of longan (*Dimocarpus longan* Lour. Fen Ke) seed extract. Int. J. Food Sci. Nutr. 65, 589–593. 10.3109/09637486.2014.886181 24533783

[B74] Ustuner O. Anlas C. Bakirel T. Ustun-Alkan F. Diren Sigirci B. Ak S. (2019). *In vitro* evaluation of antioxidant, anti-inflammatory, antimicrobial and wound healing potential of *Thymus Sipyleus* Boiss. Subsp. *Rosulans* (Borbas) *Jalas* . Molecules 24, 3353. 10.3390/molecules24183353 31540139 PMC6767006

[B75] Wang H. R. Sui H. C. Ding Y. Y. Zhu B. T. (2018). Stimulation of the production of prostaglandin E₂ by ethyl gallate, a natural phenolic compound richly contained in longan. Biomolecules 8, 91. 10.3390/biom8030091 30200641 PMC6165217

[B76] WFO (2022). Caesalpinia mimosoidesLam. Published on the internet Available at: http://www.worldfloraonline.org/taxon/wfo-0000196945 (Accessed August 27, 2022).

[B77] World Health Organization (2013). WHO traditional medicine strategy: 2014-2023. Available at: https://apps.who.int/iris/handle/10665/92455 .

[B78] Yadav E. Singh D. Yadav P. Verma A. (2018). Antioxidant and anti-inflammatory properties of *Prosopis cineraria* based phenolic rich ointment in wound healing. Biomed. Pharmacother. 108, 1572–1583. 10.1016/j.biopha.2018.09.180 30372859

[B79] Youn J. S. Kim Y. J. Na H. J. Jung H. R. Song C. K. Kang S. Y. (2018). Antioxidant activity and contents of leaf extracts obtained from*Dendropanax morbifera* LEV are dependent on the collecting season and extraction conditions. Food Sci. Biotechnol. 28, 201–207. 10.1007/s10068-018-0352-y 30815311 PMC6365350

