# Supplementary material for: Ethyl gallate isolated from phenol-enriched fraction of Caesalpinia mimosoides Lam. Promotes cutaneous wound healing: a scientific validation through bioassay-guided fractionation
Source: Front Pharmacol. 2023 Jun 16;14:1214220. doi: 10.3389/fphar.2023.1214220 (PMC10311562; doi:10.3389/fphar.2023.1214220)
Supplement: Supplementary file 1 [file Table1.pdf]

SUPPLEMENTARY TABLE S1

Yield of fractions and sub fractions

Fractions	Yield (mg/g)	Yield (%)	Sub fractions	Yield (mg/g)	Yield (%)
Fraction 1	8.54	0.85	Sub-fraction 9.1	29.98	3.00
Fraction 2	5.70	0.57	Sub-fraction 9.2	15.25	1.52
Fraction 3	6.94	0.69	Sub-fraction 9.3	37.64	3.76
Fraction 4	8.06	0.81	Sub-fraction 9.4	45.37	4.54
Fraction 5	8.44	0.84	Sub-fraction 9.5	38.24	3.82
Fraction 6	3.01	0.30	Sub-fraction 9.6	26.11	2.61
Fraction 7	4.66	0.47	Sub-fraction 9.7	41.10	4.11
Fraction 8	16.84	1.68	Sub-fraction 9.8	54.78	5.48
Fraction 9	202.13	20.21	Sub-fraction 9.9	25.07	2.51
Fraction 10	9.05	0.90	Sub-fraction 9.10	46.07	4.61
Fraction 11	6.60	0.66	Sub-fraction 9.11	38.39	3.84
Fraction 12	91.08	9.11	Sub-fraction 9.12	42.05	4.20
Fraction 13	85.10	8.51	Sub-fraction 9.13	378.11	37.81
Fraction 14	47.04	4.70	Sub-fraction 9.14	80.84	8.08
Fraction 15	67.30	6.73	Sub-fraction 9.15	100.99	10.10
Fraction 16	75.72	7.57	EG	742.86	74.29
Fraction 17	42.07	4.21			
Fraction 18	12.40	1.24			
Fraction 19	47.31	4.73			
Fraction 20	41.28	4.13			
Fraction 21	43.05	4.31			
Fraction 22	55.40	5.54			
Fraction 23	56.94	5.69			
Fraction 24	55.36	5.54			
