# Supplementary material for: Ethyl gallate isolated from phenol-enriched fraction of Caesalpinia mimosoides Lam. Promotes cutaneous wound healing: a scientific validation through bioassay-guided fractionation
Source: Front Pharmacol. 2023 Jun 16;14:1214220. doi: 10.3389/fphar.2023.1214220 (PMC10311562; doi:10.3389/fphar.2023.1214220)
Supplement: Supplementary file 2 [file Table2.pdf]

**SUPPLEMENTARY TABLE S2**

Antioxidant activity ($IC_{50}$) of fraction 9, sub fraction 9.13, EG and reference standards

| Samples | $IC_{50}$ (µg/ml) | |
|---|---|---|
| | DPPH | ABTS |
| Fraction 9 | $19.69 \pm 0.57^{a}$ | $26.47 \pm 2.12^{a}$ |
| Sub fraction 9.13 | $11.65 \pm 0.69^{b}$ | $17.15 \pm 0.62^{b,c}$ |
| EG | $8.83 \pm 0.71^{c}$ | $15.99 \pm 0.33^{c}$ |
| Standard gallic acid | $8.33 \pm 0.41^{c}$ | - |
| Standard catechin | - | $15.12 \pm 0.42^{c}$ |

Different letters ($^{a-c}$) in each column indicate significant differences among the variables by Tukey Post hoc analysis at $p \leq 0.05$
