# Supplementary material for: Ethyl gallate isolated from phenol-enriched fraction of Caesalpinia mimosoides Lam. Promotes cutaneous wound healing: a scientific validation through bioassay-guided fractionation
Source: Front Pharmacol. 2023 Jun 16;14:1214220. doi: 10.3389/fphar.2023.1214220 (PMC10311562; doi:10.3389/fphar.2023.1214220)
Supplement: Supplementary file 6 [file Image1.pdf]

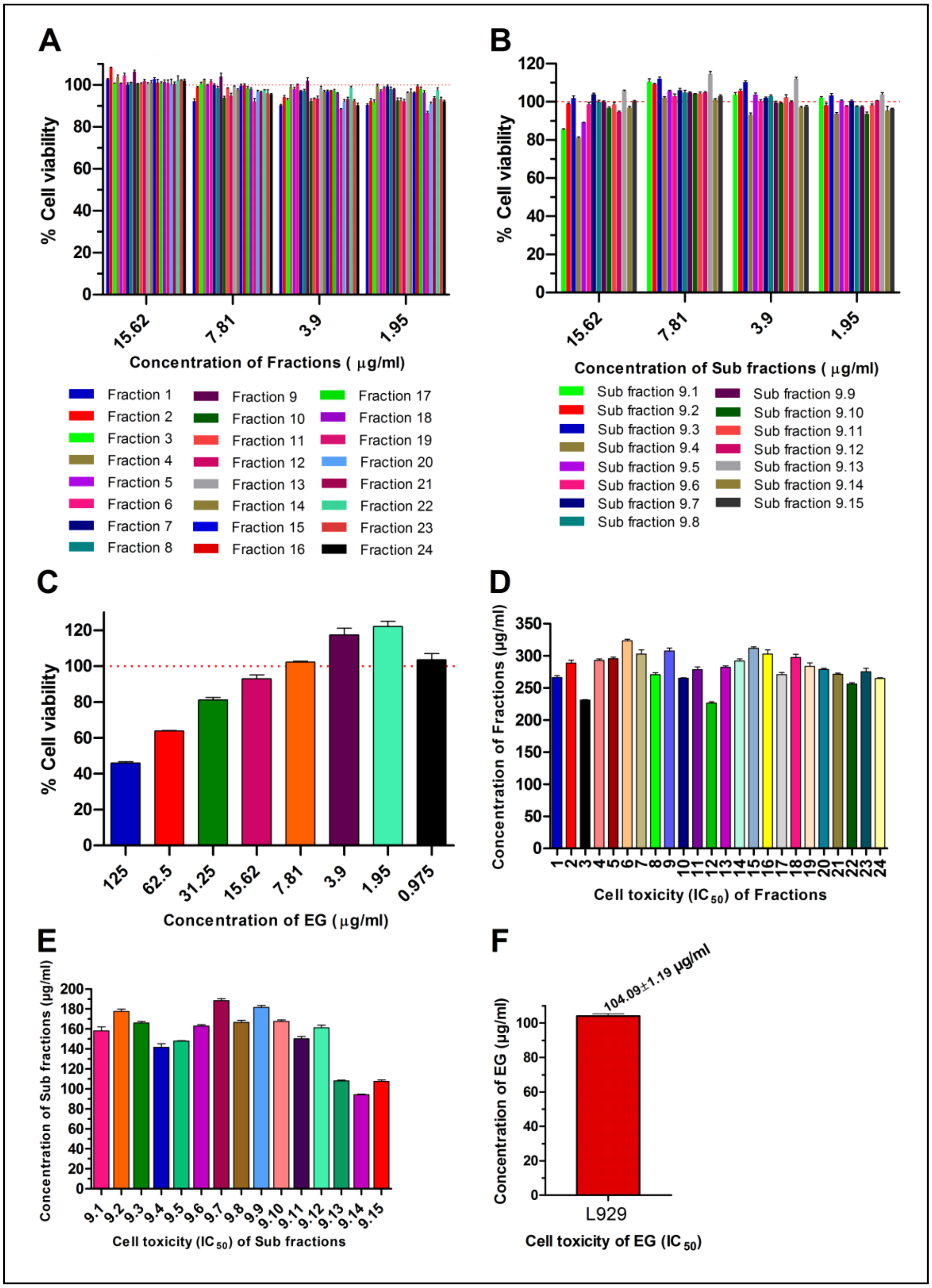

SUPPLEMENTARY FIGURE S1

Percentage cell viability and cell toxicity (IC_{50}) of fractions, sub-fractions and EG. **(A)** Percentage cell viability of 24 fractions. **(B)** Percentage cell viability of 15 sub-fractions. **(C)** Percentage cell viability of EG. **(D)** Cell toxicity (IC_{50}) of 24 fractions. **(E)** Cell toxicity (IC_{50}) of 15 sub-fractions. **(F)** Cell toxicity (IC_{50}) of EG.
